# Expression of a Constitutively Active Form of *Hck* in Chondrocytes Activates Wnt and Hedgehog Signaling Pathways, and Induces Chondrocyte Proliferation in Mice

**DOI:** 10.3390/ijms21082682

**Published:** 2020-04-12

**Authors:** Viviane K. S. Kawata Matsuura, Carolina Andrea Yoshida, Hisato Komori, Chiharu Sakane, Kei Yamana, Qing Jiang, Toshihisa Komori

**Affiliations:** 1 Basic and Translational Research Center for Hard Tissue Disease, Nagasaki University Graduate School of Biomedical Sciences, Nagasaki 852-8588, Japan; 2 Division of Comparative Medicine, Life Science Support Center, Nagasaki University, Nagasaki 852-8523, Japan; 3 Teijin Institute for Bio-Medical Research, TEIJIN LIMITED, Tokyo 100-8585, Japan

**Keywords:** Hck, Runx2, chondrocyte proliferation, Wnt, hedgehog

## Abstract

Runx2 is required for chondrocyte proliferation and maturation. In the search of Runx2 target genes in chondrocytes, we found that Runx2 up-regulated the expression of hematopoietic cell kinase (*Hck)*, which is a member of the Src tyrosine kinase family, in chondrocytes, that *Hck* expression was high in cartilaginous limb skeletons of wild-type mice but low in those of *Runx2*^–/–^ mice, and that Runx2 bound the promoter region of *Hck*. To investigate the functions of Hck in chondrocytes, transgenic mice expressing a constitutively active form of *Hck* (*Hck*^CA^) were generated using the *Col2a1* promoter/enhancer. The hind limb skeletons were fused, the tibia became a large, round mass, and the growth plate was markedly disorganized. Chondrocyte maturation was delayed until E16.5 but accelerated thereafter. BrdU-labeled, but not terminal deoxynucleotidyl transferase-mediated dUTP nick end labeling (TUNEL)-positive, chondrocytes were increased. Furthermore, *Hck* knock-down reduced the proliferation of primary chondrocytes. In microarray and real-time RT-PCR analyses using hind limb RNA from *Hck*^CA^ transgenic mice, the expression of Wnt (*Wnt10b*, *Tcf7*, *Lef1*, *Dkk1*) and hedgehog (*Ihh*, *Ptch1*, and *Gli1*) signaling pathway genes was upregulated. These findings indicated that *Hck*, whose expression is regulated by Runx2, is highly expressed in chondrocytes, and that *Hck*^CA^ activates Wnt and hedgehog signaling pathways, and promotes chondrocyte proliferation without increasing apoptosis.

## 1. Introduction

In the process of endochondral ossification, mesenchymal cells condensate and acquire the phenotype of chondrocytes to produce Col2a1 and proteoglycan. Sox9 is required for the mesenchymal cell condensation, and Sox9, Sox5, and Sox6 induce *Col2a1* expression [1,2]. Chondrocytes proliferate and differentiate into prehypertrophic chondrocytes, which express *Pthr1* and *Ihh*, and further differentiate into hypertrophic chondrocytes, which express *Col10a1*. Hypertrophic chondrocytes terminally differentiate into terminal hypertrophic chondrocytes, which express *Mmp13*, *Ibsp*, and *Spp1*. In long bones, differentiation and proliferation are organized, and the growth plate, which is composed of resting, proliferating, prehypertrophic, hypertrophic, and terminal hypertrophic chondrocyte layers, is formed. Vascular invasion occurs in the layer of terminal hypertrophic chondrocytes, the terminal hypertrophic chondrocytes transdifferentiate to osteoblastic lineage cells or die by apoptosis, osteoblast lineage cells invade from the perichondrium, and cartilage is replaced with bone [3].

Runx2 is a transcription factor that belongs to Runx family proteins, and is essential for bone development and osteoblast differentiation [4,5]. Runx2 is also expressed in resting and proliferating chondrocyte layers, and its expression is up-regulated in prehypertrophic chondrocytes [6,7]. Runx2 is also essential for the maturation of chondrocytes in the resting and proliferating layers to those in the hypertrophic layer [8,9,10,11]. Runx3 is partly involved in this maturation [11]. Runx2 is also required for chondrocyte proliferation, as it is markedly reduced in the cartilaginous skeleton of *Runx2*^–/–^ mice. Runx2 directly regulates the expression of *Ihh*, which induces chondrocyte proliferation [11,12]. Ihh induces *Pthlh* expression and Pthlh reduces *Runx2* expression through Pthr1, forming a negative feedback loop for chondrocyte maturation [13,14,15].

Although *Ihh* is one of the target genes of Runx2, we further searched Runx2 target genes for chondrocyte maturation and proliferation by introducing *Runx2* into *Runx2*^–/–^ primary chondrocytes. We found that the expression of hematopoietic cell kinase (*Hck*) is induced by Runx2. Hck is a non-receptor protein tyrosine kinase, which belongs to the Src protein tyrosine kinase family. Hck expression is elevated in several types of leukemia, and Hck contributes to leukemogenesis by enhancing cell proliferation and survival through the association with the oncogenic fusion proteins BCR/ABL and TEL/ABL [16]. Although Hck has been reported to be expressed in myeloid and B-lymphocyte lineages [17], our results showed that it is highly expressed in chondrocytes. As Src family proteins have redundant functions and *Hck*^–/–^ mice exhibit minimal phenotypes [18,19], we generated transgenic mice expressing a constitutively active form of *Hck* (*Hck*^CA^) in chondrocytes to investigate the functions of Hck in chondrocytes. The expression of *Hck*^CA^ expanded the cartilage by increasing chondrocyte proliferation, but disrupted the organization of the growth plate.

## 2. Results

### 2.1. Hck Expression Was Regulated by Runx2 in Chondrocytes

**Figure 1:**
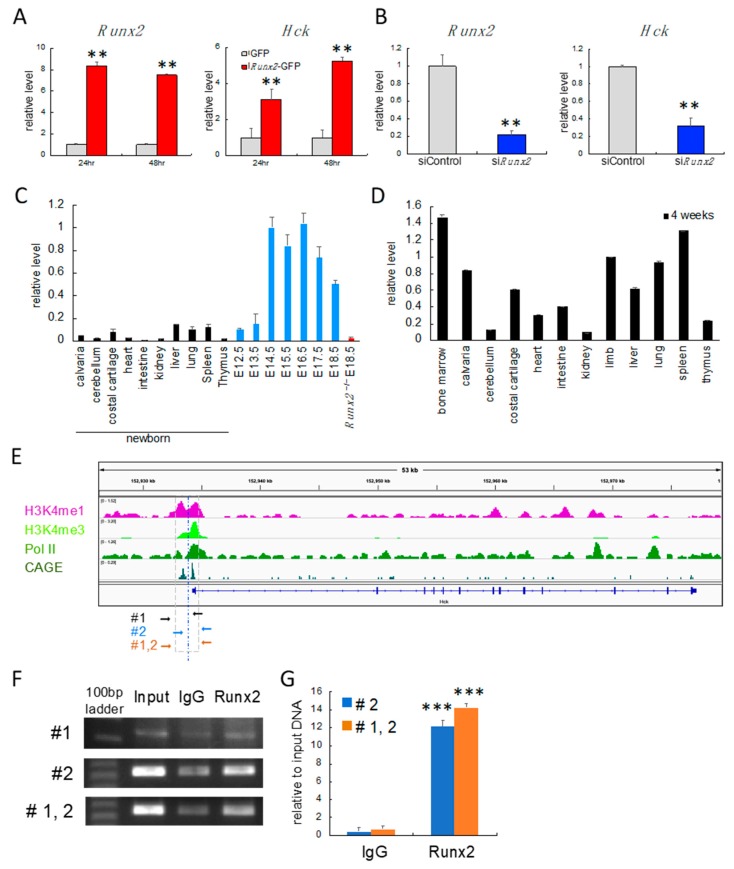
Real-time RT-PCR and chromatin-immunoprecipitation (ChIP) analyses. (**A**–**D**) Real-time RT-PCR analyses. **A**, Induction of *Hck* expression by Runx2. Wild-type primary chondrocytes were infected with adenovirus expressing GFP (grey columns) or *Runx2* and GFP (red columns). The values in GFP-expressing cells were defined as 1, and relative levels are shown. *n* = 4. **B**, Reduction of *Hck* by siRNA for *Runx2*. Primary chondrocytes were infected with siControl or si*Runx2*. The values of siControl were defined as 1, and relative levels are shown. *n* = 4. ** *p* < 0.01. **C**, Abundance of *Hck* transcripts in tissues of newborn mice, limb tissue in wild-type embryos at E12.5−18.5, and *Runx2*^−/−^ embryos at E18.5. **D**, Abundance of *Hck* transcripts in tissues at 4 weeks of age. Three mice were analyzed for each tissue. The values in limbs at E14.5 (**C**) and 4 weeks of age (**D**) were set as 1, and relative levels are shown. (**E**) Genome browser views of H3K4me1, H3K4me3, and Pol II ChIP-seq, and cap analysis of gene expression (CAGE) profiles in limbs are shown. A yellow dashed box highlights the region of the transcription start site (TSS) of *Hck*. Two TSS peaks in the CAGE were correctly mapped to the known 5′ end in the RefSeq of *Hck*. The locations of the primer sets (#1, #2, or #1 and 2) used in **F** and **G** are shown on the bottom. High-throughput ChIP-seq data sets were obtained from the ENCODE website. (**F** and **G**) ChIP analysis using ATDC5 cells. DNA before immunoprecipitation (input) and after immunoprecipitation with anti-Runx2 antibody or mouse IgG was amplified by PCR (**F**) and real-time PCR (**G**) using primers shown in **E**. Three independent ChIP assays were performed and the means ± SD are shown in G. *Versus IgG. *** *p* < 0.001.

Overexpression of Runx2 induced *Hck* expression in primary chondrocytes, and the transfection of *Runx2* siRNA reduced *Hck* expression (Figure 1A,B). *Hck* was highly expressed in the liver, spleen, and costal cartilage in newborn mice (Figure 1C). In the embryonic stage, *Hck* expression in limbs was up-regulated at E14.5 and mildly down-regulated after E17.5, and its expression was much higher than that in the liver, spleen, and costal cartilage in newborn mice (Figure 1C). However, *Hck* expression in the limbs of *Runx2*^–/–^ mice at E18.5, in which the skeletons were composed of cartilaginous tissues, was much lower than that in wild-type embryos (Figure 1C). *Hck* was highly expressed in bone marrow, calvaria, costal cartilage, limb, liver, lung, and spleen at 4 weeks of age (Figure 1D). 

In cap analysis of gene expression (CAGE), there were two transcription start sites at the 5’ end of *Hck* exon 1 (#1 and #2) (Figure 1E). We performed chromatin-immunoprecipitation (ChIP) analysis using Runx2 antibody. The precipitated DNA was amplified by PCR using the primers covering #1, #2, or #1 and #2 (Figure 1E,F). Significant amplification was obtained using the primers covering #2 or #1 and #2. We also confirmed the specific binding of Runx2 antibody to the region covering #2 by real-time RT-PCR (Figure 1G). We did not detect significant amplification by real-time RT-PCR using primers covering #1 only.

### 2.2. The Morphology of the Lower Limbs Was Markedly Disturbed, Forming a Large Mass, and the Process of Endochondral Ossification Was Delayed in Hck^CA^ Transgenic (tg) Embryos at E16.5

**Figure 2:**
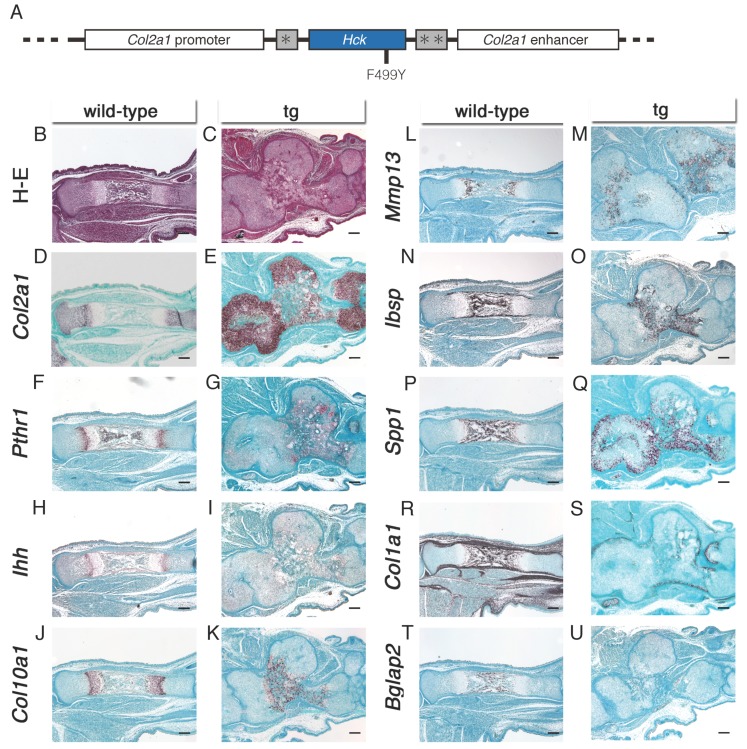
Histological analyses of tibiae in *Hck*^CA^ tg embryos at E16.5. (**A**) Diagram of the DNA construct used to generate tg embryos that express *Hck*^CA^ under the control of the mouse *Col2a1* promoter-enhancer. The tyrosine at amino acid 499 was substituted with phenylalanine in *Hck*^CA^. * Intron from rabbit β-globin gene, ** Polyadenylation signal from SV40. (**B**,**C**) Hematoxylin and eosin (**H**–**E**) staining of wild-type and *Hck*^CA^ tg embryos. (**D**–**U**) In situ hybridization using *Col2a1* (**D**,**E**), *Pthr1* (**F**,**G**), *Ihh* (**H**,**I**), *Col10a1* (**J**,**K**), *Mmp13* (**L**,**M**), *Ibsp* (**N**,**O**), *Spp1* (**P**,**Q**), *Col1a1* (**R**,**S**), and *Bglap2* (**T**,**U**) probes in wild-type (**B**,**D**,**F**,**H**,**J**,**L**,**N**,**P**,**R**,**T**) and *Hck*^CA^ tg (**C**,**E**,**G**,**I**,**K**,**M**,**O**,**Q**,**S**,**U**) embryos. Scale bars: 200 μm.

Tg mice, which express *Hck*^CA^ in chondrocytes, were generated by inserting *Hck* cDNA with a tyrosine-to-phenylalanine mutation at amino acid 499 into the *Col2a1* promoter-enhancer construct (Figure 2A). As mice died after birth, they were analyzed at F_0_. The numbers of tg embryos obtained at E14.5−18.5 are shown in Table S1.

Tibiae were histologically compared at E16.5. Bone marrow was formed in wild-type embryos, whereas the tibiae and fibulae were fused and formed a large, highly deformed mass in *Hck*^CA^ tg embryos, and bone marrow formation was not evident (Figure 2B,C). *Col2a1* expression was detected in resting, proliferating, and prehypertrophic chondrocyte layers in the epiphysis, and *Col10a1* expression was detected in the hypertrophic chondrocyte layer in the metaphysis in wild-type embryos. On the other hand, *Col2a1* expression was detected in the periphery of the mass and *Col10a1* expression was detected in hypertrophic chondrocytes in the center of the mass in *Hck*^CA^ tg embryos (Figure 2D,E,J,K). *Pthr1* and *Ihh* expression was detected in the prehypertrophic chondrocyte layer in wild-type embryos, whereas their expression was detected in the region surrounding *Col10a1*-positive hypertrophic chondrocytes in *Hck*^CA^ tg embryos (Figure 2F–I). *Mmp13*, *Ibsp*, and *Spp1* expression was noted in the terminal hypertrophic chondrocyte layer, and *Ibsp* and *Spp1* expression was also noted in osteoblasts in the bone marrow and bone collar in wild-type embryos, whereas *Mmp13* and *Ibsp* expression was detected in terminal hypertrophic chondrocytes at the center of the cartilaginous mass, and *Spp1* expression was detected in the large area of the cartilaginous mass in *Hck*^CA^ tg embryos (Figure 2L–Q). *Spp1* expression in immature chondrocytes was also evident in *Hck*^CA^ tg embryos at E14.5. Although *Ibsp* expression was restricted to terminal hypertrophic chondrocytes, *Col2a1*-expressing immature chondrocytes also expressed *Spp1* in *Hck*^CA^ tg embryos at E14.5 (Figure S1). *Col1a1* expression was detected in osteoblasts in the bone marrow and bone collar in wild-type embryos, whereas it was detected in the restricted region at the center of the cartilaginous mass and in the perichondrium in *Hck*^CA^ tg embryos at E16.5 (Figure 2R,S). The expression of *Bglap2*, which is expressed in mature osteoblasts [20], was detected in osteoblasts in the bone collar in wild-type embryos, whereas it was virtually absent in *Hck*^CA^ tg embryos (Figure 2T,U). The delay in osteoblast differentiation was likely caused by the decelerated chondrocyte maturation and disorganized growth plate because *Ihh* expression in prehypertrophic chondrocytes is essential for osteoblast differentiation in endochondral bones [12,21].

### 2.3. Limb Bones Were Short and Thick, and They Were Fused in Hck^CA^ tg Embryos at E18.5

**Figure 3:**
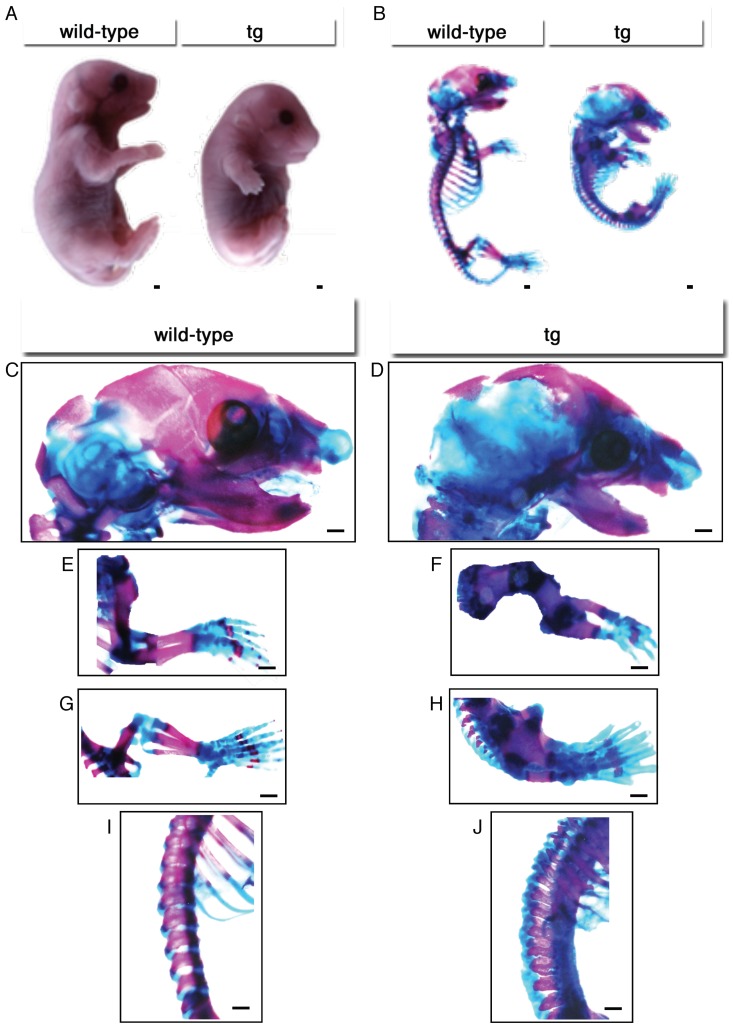
Skeletal system in *Hck*^CA^ tg embryos at E18.5. (**A**) Physical appearance of wild-type and *Hck*^CA^ tg embryos. (**B**–**J**) Skeletal system. The whole skeletons (**B**), and enlarged views of the head (**C**,**D**), forelimbs (**E**,**F**), hind limbs (**G**,**H**), and vertebrae (**I**,**J**) in wild-type and *Hck*^CA^ tg embryos. Scale bars: 1.25 mm.

*Hck*^CA^ tg embryos exhibited dwarfism and had short limbs at E18.5 (Figure 3A). In skeletal preparation, mineralization in *Hck*^CA^ tg embryos progressed to the level of wild-type mice, although the cartilaginous nasal capsule and the cartilaginous part of the interparietal bone were enlarged, and ossification of the exoccipital bone and basioccipital bone was delayed compared to that in wild-type embryos (Figure 3B–D). In the forelimb, the humerus, radius, and ulna were shorter and thicker than those in wild-type embryos, and were fused (Figure 3E,F). The metacarpal bones and phalanxes were thick but less mineralized than those in wild-type embryos (Figure 3E,F). In the hind limb, the femur and tibia were shorter and thicker than those in wild-type embryos, and they were fused, and the tibia became a large round mass (Figure 3G,H). The metatarsal bones and phalanges were thick but less mineralized than those in wild-type embryos (Figure 3G,H). Furthermore, the vertebral bodies were fused (Figure 3I,J).

### 2.4. Most of the Cartilaginous Mass Was Replaced With Bone, But Osteoblast Differentiation Was Still Delayed in Hck^CA^ tg Embryos at E18.5

**Figure 4:**
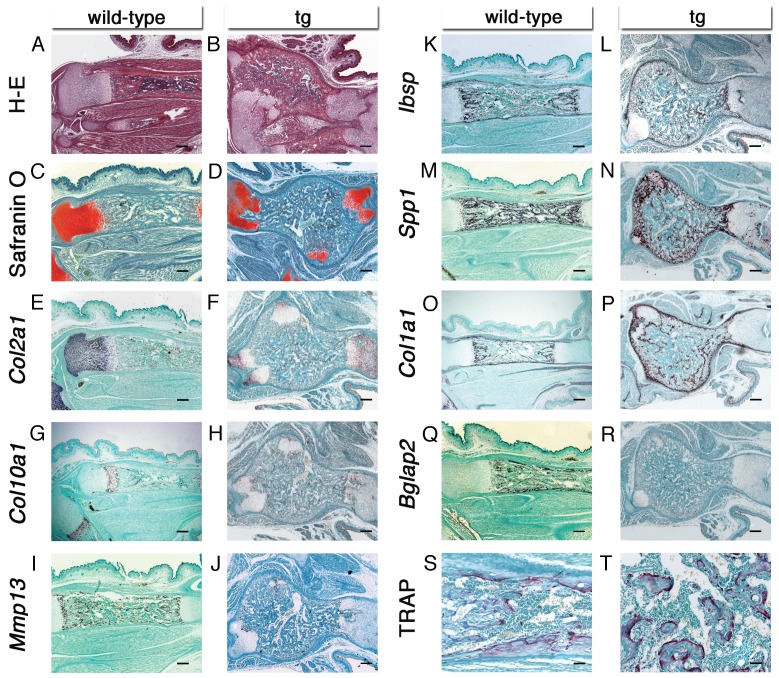
Histological analyses of tibiae in *Hck*^CA^ tg embryos at E18.5. (**A** and **B**) H-E staining in wild-type (**A**) and *Hck*^CA^ tg (**B**) embryos. (**C** and **D**) Safranin O staining in wild-type (**C**) and *Hck*^CA^ tg (**D**) embryos. (**E**–**R**) *In situ* hybridization using *Col2a1* (**E**,**F**), *Col10a1* (**G**,**H**), *Mmp13* (**I**,**J**), *Ibsp* (**K**,**L**), *Spp1* (**M**,**N**), *Col1a1* (**O**,**P**), and *Bglap2* (**Q**,**R**) probes in wild-type (**A**,**C**,**E**,**G**,**J**,**K**,**M**,**O**,**Q**) and *Hck*^CA^ tg (**B**,**D**,**F**, **H**,**J**,**L**,**N**,**P**,**R**) embryos. (**S** and **T**) TRAP staining in wild-type (**S**) and *Hck*^CA^ tg (**T**) embryos. Scale bars: 200 μm.

Histological analysis demonstrated that the tibiae were largely replaced with bone and bone marrow. The safranin O-positive growth plate was composed of *Col2a1*-positive chondrocytes and *Col10a1*-positive hypertrophic chondrocytes. In addition, *Mmp13*, *Ibsp*, and *Spp1* expression was detected in terminal hypertrophic chondrocytes, and *Ibsp*-positive, *Spp1*-positive, *Col1a1*-positive, or *Bglap2*-positive osteoblasts were abundant in the bone marrow and bone collar in wild-type embryos (Figure 4A,C,E,G,I,K,M,O,Q). In *Hck*^CA^ tg embryos, the tibiae were largely replaced with bone and bone marrow, and the remaining safranin O-positive cartilaginous regions were composed of *Col2a1*-positive or *Col10a1*-positive chondrocytes (Figure 4B,D,F,H). Furthermore, *Mmp13*, *Ibsp*, and *Spp1* expression was detected in the terminal hypertrophic chondrocytes at the border of bone marrow and cartilage, although some *Col2a1*-positive chondrocytes were also *Spp1*-positive (Figure 4F,J,L,N). *Ibsp*, *Spp1*, and *Col1a1* expression was detected in osteoblasts in the bone marrow and the periphery of the mass, whereas *Bglap2* expression was virtually absent in *Hck*^CA^ tg embryos (Figure 4L,N,P,R). Tartrate-resistant acid phosphatase (TRAP)-positive osteoclasts were similarly observed in wild-type and *Hck*^CA^ tg embryos (Figure 4S,T).

### 2.5. BrdU Uptake Was Increased in Hck^CA^ tg Embryos, but the Frequency of Terminal Deoxynucleotidyl Transferase-Mediated dUTP Nick End Labeling (TUNEL)-Positive Cells Was Similar to That in Wild-Type Embryos

**Figure 5:**
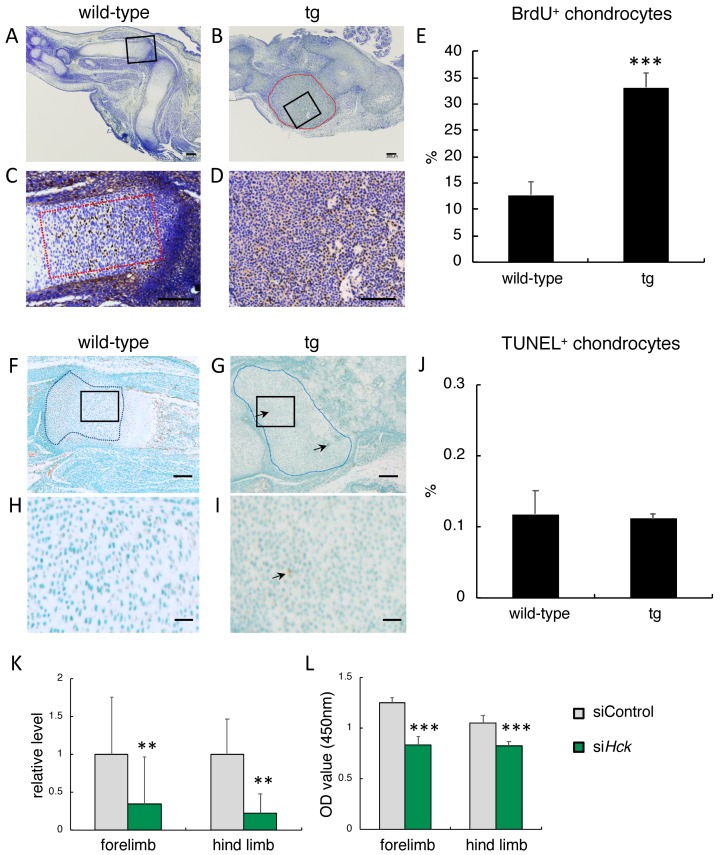
BrdU and TUNEL staining in *Hck*^CA^ tg embryos and knock-down experiments in vitro. (**A**–**D**) BrdU staining in wild-type (**A**,**C**) and *Hck*^CA^ tg (**B**,**D**) embryos at E14.5. The black boxed regions in **A** and **B** are magnified in **C** and **D**, respectively. (**E**) The frequencies of BrdU-positive cells. The BrdU-positive cells and total number of chondrocytes in the surrounded regions by red dotted lines in **B** and **C** were counted, and the frequencies of BrdU-positive cells are shown. (**F**–**I**) TUNEL staining in wild-type (**F**,**H**) and *Hck*^CA^ tg (**G**,**I**) embryos at E16.5. The boxed regions in **F** and **G** were magnified in **H** and **I**, respectively. The arrows in **G** and **I** indicate TUNEL-positive cells. (**J**) The frequencies of TUNEL-positive cells. The TUNEL-positive cells and total number of chondrocytes in the surrounded regions by blue dotted lines in **F** and **G** were counted, and the frequencies of TUNEL-positive cells are shown. Three mice were analyzed, and the means ± SD are shown in **E** and **J**. *** *p* < 0.001. Scale bars: 200 μm (**A**–**D**,**F**,**G**), 50 μm (**H**,**I**). (**K** and **L**) Knock-down experiments using primary chondrocytes from forelimbs or hind limbs. (**K**) Real-time RT-PCR analysis of *Hck* expression in primary chondrocytes transfected with control siRNA or *Hck* siRNA. (**L**) Cell number (OD value) 72 hours after the transfection of control siRNA or *Hck* siRNA. Three independent experiments showed similar results and the representative data are shown. ** *p* < 0.01, *** *p* < 0.001.

As the limb skeletons were thick and the tibiae were a large cartilaginous mass in *Hck*^CA^ tg embryos at E14.5 and E16.5, chondrocyte proliferation was examined by BrdU labeling at E14.5 (Figure 5A–D). The frequencies of BrdU-positive chondrocytes in *Hck*^CA^ tg embryos were much higher than those in wild-type embryos (Figure 5E). We also examined the frequencies of TUNEL-positive cells at E16.5 because abnormal stimulation of the cell cycle induces apoptosis [22] (Figure 5F–I). The frequencies of TUNEL-positive cells were similar between wild-type and *Hck*^CA^ tg embryos (Figure 5J).

### 2.6. Knock-Down of Hck Expression Reduced the Proliferation of Primary Chondrocytes

To investigate whether Hck is involved in the proliferation of chondrocytes, primary chondrocytes isolated from forelimbs or hind limbs were transfected with *Hck* siRNA or control siRNA. *Hck* expression in primary chondrocytes transfected with *Hck* siRNA was one third to one fourth of that in those transfected with control siRNA (Figure 5K). Treatment with *Hck* siRNA significantly reduced the proliferation of primary chondrocytes derived from either forelimbs or hind limbs compared with control siRNA treatment (Figure 5L).

### 2.7. Microarray Analysis Demonstrated that the Expression of Wnt and Hedgehog Signaling Pathway Genes Were Increased in Hck^CA^ tg Embryos

**Figure 6:**
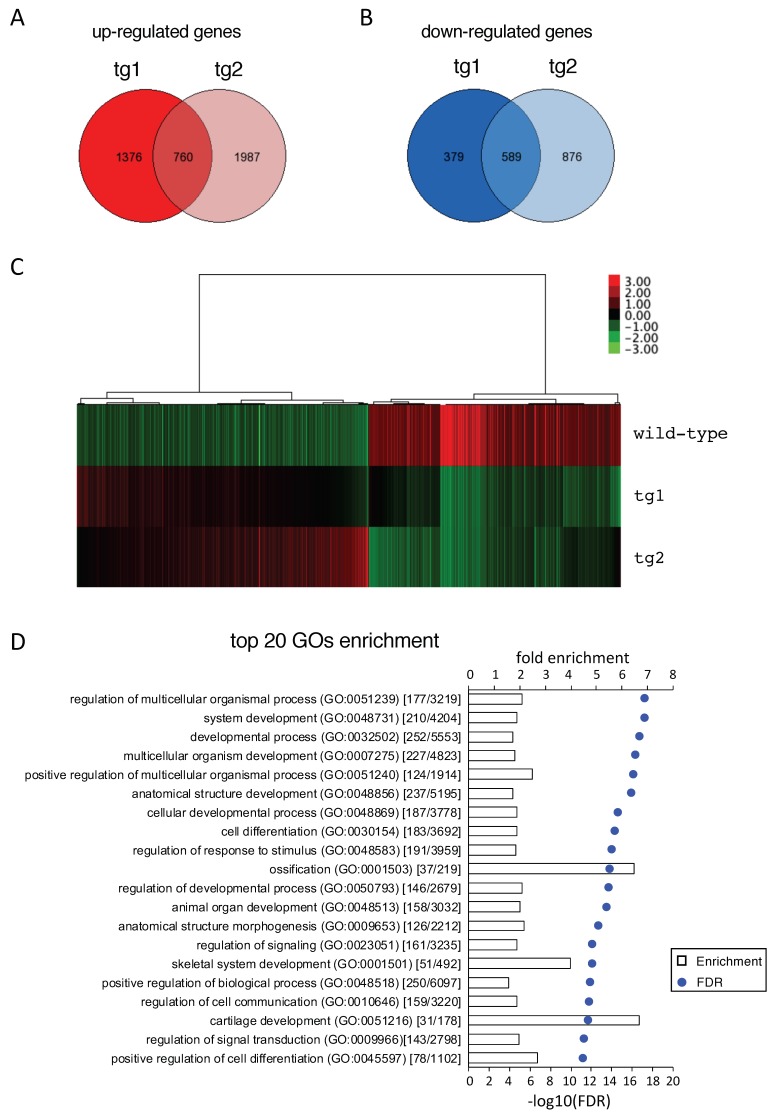
Microarray analysis. Microarray analysis was performed using one wild-type and two *Hck*^CA^ tg (tg1 and tg2) samples. (**A** and **B**) Venn diagrams were drawn based on our microarray data sets. Red circles indicate the numbers of genes up-regulated in the tg group (vs. wild-type group, fold change ≥ 1.5) (**A**), and blue circles represent the numbers of down-regulated genes in the tg group (vs. wild-type group, fold change < 0.5) (**B**). The numbers in the overlapped regions reflect up-regulated or down-regulated genes in both tg1 and tg2 embryos compared with the gene expression in wild-type embryos. (**C**) Heatmap image of microarray data of the overlapped genes in **A** and **B**. Green indicates reduced expression and red indicates increased expression. (**D**) Top 20 of gene ontology (GO) enrichment (false discovery rate (FDR) < 0.05) of differentially expressed genes are displayed in an ascending order of FDR. The numbers in the parentheses represent the gene count of matched term per number of genes in the reference list.

**Figure 7:**
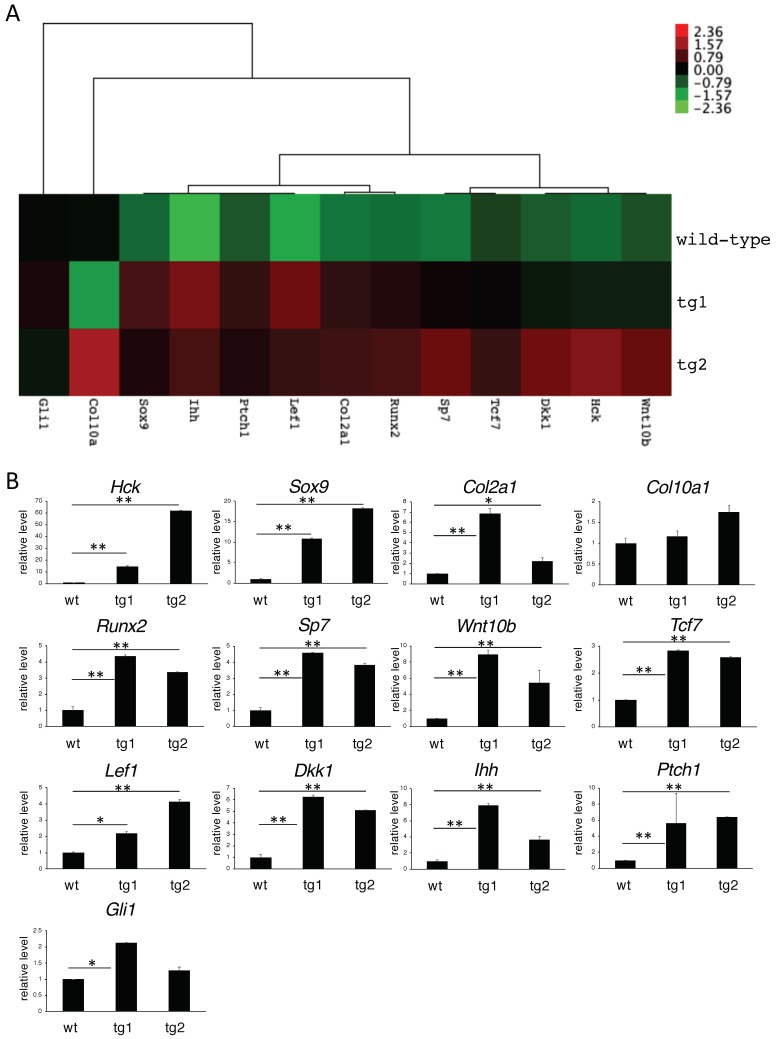
Heatmap and real-time RT-PCR analysis of the selected genes. (**A**) Heatmap of the selected genes. (**B**) Real-time RT-PCR analysis of the selected genes was performed using the same RNA samples used for microarray. The values in wild-type (wt) embryos were defined as 1, and relative levels are shown. All quantitative data were obtained from three independent experiments and the means ± SD are shown. Versus wild-type sample, * *p* < 0.05, ** *p* < 0.01.

To reveal the mechanism of increased chondrocyte proliferation in *Hck*^CA^ tg embryos, microarray analysis was performed using a wild-type RNA and two tg RNAs extracted from the hind limbs at E15.5. Seven hundred and sixty genes were up-regulated in both tg embryos (tg1 and tg2) and 589 genes were down-regulated in both tg embryos compared with wild-type embryos (Figure 6A–C). We focused on the up-regulated genes because gene ontology (GO) terms related to skeletal development were enriched in the up-regulated genes, but not in the down-regulated genes, in *Hck*^CA^ tg embryos compared with wild-type embryos (Figure 6D and data not shown). We focused on the genes in the top 20 enriched GO terms (Figure 6D and Table S2). The expression of *Sox9* and *Col2a1* was increased on microarray and real-time RT-PCR analyses (Figure 7). Moreover, *Runx2* and *Sp7* expression, which is up-regulated in prehypertrophic chondrocytes, was increased in *Hck*^CA^ tg embryos compared to wild-type embryos (Figure 7). However, *Col10a1* expression was not significantly increased in tg embryos (Figure 7). Therefore, immature and prehypertrophic chondrocytes were enriched in *Hck*^CA^ tg embryos at E15.5. This is consistent with the phenotype in *Hck*^CA^ tg embryos at E14.5 and E16.5, in which chondrocyte maturation was delayed and most of the hind limb skeletons were still cartilaginous (Figure 2 and Figure S1). The expression of Wnt signaling pathway genes (*Wnt10b*, *Tcf7*, *Lef1*, and *Dkk1*) and hedgehog signaling pathway genes (*Ihh* and *Ptch1*) was increased on the microarray and real-time RT-PCR analyses. *Gli1* expression was also up-regulated according to real-time RT-PCR analysis, although it was not significantly up-regulated on microarray analysis (Figure 7). As *Tcf7* and *Dkk1* expression is induced by the Wnt signaling pathway, and *Ptch1* and *Gli1* expression is induced by the hedgehog signaling pathway [23], both signaling pathways were considered to be activated in *Hck*^CA^ tg embryos.

## 3. Discussion

*Hck* was highly expressed in cartilaginous skeletons, especially in limb skeletons of embryos, and its expression was regulated by Runx2. In *Hck*^CA^ tg embryos, the forelimb and hind limb skeletons and vertebral bodies were fused, and the tibiae became a large round mass. Chondrocyte maturation and vascular invasion into the cartilage were delayed. However, the replacement of the cartilage with bone was accelerated after vascular invasion into the cartilage. The large cartilaginous mass was formed through the increased chondrocyte proliferation without inducing apoptosis in *Hck*^CA^ tg embryos. Furthermore, knock-down of *Hck* reduced the proliferation of primary chondrocytes. Src family proteins, including Hck, promote cell proliferation through Akt, ERK, and Stat signaling pathways in cancer cells [16]. This is the first study to propose that Hck is involved in chondrocyte proliferation and that Hck promotes this by activating Wnt and hedgehog signaling pathways.

As Src family proteins have redundant functions, *Hck*^–/–^*Fgr*^–/–^ double knockout mice are overtly normal [18]. Therefore, it is difficult to evaluate the physiological functions of Hck in chondrocytes. Runx2 is required for chondrocyte proliferation [11]—it regulates *Hck* expression in chondrocytes, and *Hck* was highly expressed in cartilaginous limb skeletons. Therefore, Hck is likely to be physiologically involved in chondrocyte proliferation. Although *Runx2* expression is up-regulated in prehypertrophic chondrocytes, it is also expressed in resting and proliferating chondrocyte layers [6,7,24]. Therefore, the induction of *Hck* expression by Runx2 in resting and proliferating chondrocytes may lead to chondrocyte proliferation. Furthermore, the induction of *Wnt10b* and *Ihh* expression by Hck in prehypertrophic chondrocytes will promote chondrocyte proliferation in the proliferating chondrocyte layer. Although Runx2 directly induces *Ihh* expression [11], this induction may be, at least in part, through the induction of *Hck* expression. As *Runx2* expression was increased in *Hck*^CA^ tg embryos and *Runx2* expression and that of Wnt and hedgehog signaling pathway genes are reciprocally regulated [25], Hck may enhance *Runx2* expression through the activation of Wnt and hedgehog signaling pathways. Moreover, *Hck* expression is up-regulated in different forms of leukemia, causing leukemogenesis, and a high copy number of the *Hck* gene is frequently detected in gastric cancer cell lines and gastric cancer patients [16,26]. Although there is no report on the involvement of Hck in chondrosarcoma, Hck may also play a role in the pathogenesis of chondrosarcoma.

The expression of a constitutively active form of β-catenin (CA-LEF), which is a fusion mutant protein of β-catenin and Lef1, in immature chondrocytes caused fusion of the limbs and inhibited chondrocyte maturation, whereas the expression of an activated β-catenin mutant in mature chondrocytes greatly promoted chondrocyte maturation and bone formation [27]. Although the cartilaginous mass in *Hck*^CA^ tg embryos was larger than that in CA-LEF embryos, the phenotypes in *Hck*^CA^ tg embryos resembled those in CA-LEF embryos, suggesting that an activated Wnt signaling pathway is, at least in part, involved in the disturbed limb development in *Hck*^CA^ tg embryos.

*Spp1* was expressed not only in terminal hypertrophic chondrocytes, but also in immature chondrocytes in *Hck*^CA^ tg embryos (Figure 2 and Figure S1). *Spp1* expression in immature chondrocytes was likely to have been caused by the activation of inflammatory signaling through Hck (Figure S2 and Table S3) because its expression is induced by inflammation [28,29]. As Runx2 is involved in the pathogenesis of osteoarthritis and the inflammatory processes induced by mechanical injury lead to the development of post-traumatic osteoarthritis [30,31,32,33], Hck may also be involved in the development of osteoarthritis, especially in osteophyte formation because of the capacity to promote chondrocyte proliferation.

In conclusion, the expression of *Hck*^CA^ in chondrocytes promoted chondrocyte proliferation, disturbed the organization of the growth plate, and formed a large mass in the tibiae. Chondrocyte proliferation was likely increased by the activation of Wnt and hedgehog signaling pathways by Hck, in addition to the well-known Src family protein signaling pathways through Akt, ERK, and Stat, although the mechanism of the activation of Wnt and hedgehog signaling pathways by Hck and the contribution to the chondrocyte proliferation remain to be clarified. Indeed, the physiological significance of Src family proteins in chondrocyte proliferation also requires further investigation. As *Hck* was highly expressed in chondrocytes, the involvement of Hck in the pathogenesis of chondrosarcoma and in osteophyte formation in osteoarthritis needs to be elucidated.

## 4. Materials and Methods

### 4.1. Cell Culture, Adenoviral Transfer, and Knock-Down Experiment

Primary chondrocytes were prepared from the limb skeletons of wild-type embryos at E15.5. Cells were plated in 48-well plates at a density of 7 × 10^4^/well DMEM/Nutrient Mixture F-12 Ham (Sigma-Aldrich, St. Louis, MO, USA) containing 5% fetal bovine serum (FBS) (Sigma-Aldrich) and 10 μg/mL of human transferrin (Roche Diagnostics, Mannheim, Germany). At confluency, primary chondrocytes were infected with either green fluorescent protein (GFP)-expressing or type II Runx2- and GFP-expressing adenovirus at a multiplicity of infection of 10 for 2 hrs. RNA was extracted using ISOGEN (Wako, Osaka, Japan) after 24 hours and 48 hours. In the Runx2 knock-down experiment, 2 × 10^5^ cells were subjected to electroporation of 10 pmol of siRNA for the control or *Runx2* using the neon transfection system (Invitrogen, Carlsbad, CA, USA). Tissues were taken from wild-type embryos, newborn mice, mice at 4 weeks of age, and *Runx2*^–/–^ embryos at E18.5. RNA was extracted using ISOGEN (Wako). For knock-down experiments of *Hck*, primary chondrocytes were prepared from the forelimbs or hind limbs of wild-type embryos at E12.5, suspended in Ham’s F12 medium (Sigma-Aldrich) supplemented with 5% FBS, and plated in 96-well culture plates at a concentration of 5.0 × 10^3^ cells per well. Chondrocytes were transfected with 2.5 μg of a plasmid encoding the silencer negative control siRNA (AM4611, Invitrogen) or *Hck* silencer select siRNA (43907771, Ambion) using the Lipofectamine 2000 DNA transfection system (Thermo Fisher Scientific, Waltham, MA, USA). The cell numbers were counted by detecting the absorbance at 450 nm using the cell counting kit-8 (DOJINDO, Kumamoto, Japan) 72 hours after transfection.

### 4.2. Real-Time PCR Analysis

Real-time RT-PCR (qPCR) was performed using the SYBR green or TaqMan system (Applied Biosystems). Total RNA was reverse transcribed by M-MLV reverse transcriptase (ReverTraAce, TOYOBO). Quantitative PCR was carried out using Light Cycler 480 SYBR Green I Master (Roche Diagnostics, Tokyo, Japan) and the Light Cycler 480 qPCR machine (Roche Diagnostics). SYBR green primer sets and TaqMan probes used in this study are listed in Table S4. We normalized the values obtained to those of β-actin.

### 4.3. Cap Analysis of Gene Expression (CAGE)

CAGE library preparation, sequencing, mapping, and gene expression analysis were performed in two independent experiments using limb RNA from wild-type embryos at E14.5 by DNAFORM (Yokohama, Japan). In brief, the cDNAs were synthesized from total RNA using random primers. The ribose diols in the 5’ cap structure of the RNAs were oxidized and biotinylated. The biotinylated RNA/cDNAs were collected by streptavidin beads. After removing the RNA strand using RNase I and adaptor ligation to both ends of cDNA, double-stranded cDNA libraries were constructed. The libraries were sequenced using a NextSeq 500 (Illumina). The obtained reads were mapped to the mouse mm9 genome using BWA software (v0.5.9).

### 4.4. Data Files from the ENCODE Project and Transcription Factor Binding Site Motif Analysis

ChIP-seq data of H3K4me1, H3K4me3, and Pol II, and RNA-seq data were produced by the Ren Lab at the Ludwig Institute for Cancer Research and viewed in the integrative genomics viewer. Transcription factor binding site motif analysis was performed using the JASPAR CORE database for vertebrates (2016).

### 4.5. ChIP

Chondrogenic cell line ATDC5 was cultured as previously described [8]. ATDC5 cells cultured for 15 days in differentiation medium were immediately subjected to chromatin preparation. The ChIP procedures used in this study were similar to those previously described [34,35]. Immunoprecipitation was carried out overnight using dynabeads M-280 (Invitrogen) and mouse monoclonal anti-Runx2 antibody (Santa Cruz, F2) or mouse IgG (Cell Signaling, Danvers, MA, USA). PCR was carried out in a 25-µL tube using KOD FX Neo DNA polymerase (Toyobo, Osaka, Japan). The real-time quantitative PCR assay was performed using a Roche LightCycler 480 qPCR machine with SYBR green (Roche Diagnostics). The primer sequences are shown in Table S4.

### 4.6. Generation of Hck^CA^ tg Mice

A constitutively active form of mouse *Hck* (499Tyr-Phe) DNA [36] was prepared by PCR. *Hck* (499Tyr-Phe) DNA was inserted into a *Col2a1*-based expression vector, which contains the promoter and enhancer of the mouse *Col2a1* gene, as previously described [9]. The construct insert, including *Hck* (499Tyr-Phe) with the *Col2a1* promoter/enhancer, was injected into the pronuclei of the fertilized eggs from F1 hybrid mice (C57BL/6×C3H). Genomic DNA was extracted from the liver of embryos, and incorporation of the fragments into the genome was examined by PCR. Total RNA was extracted from the upper-body skeletons, and the transgene expression was assayed by real-time RT-PCR analysis. Prior to the study, all experimental protocols were reviewed and approved by the Animal Care and Use Committee of Nagasaki University Graduate School of Biomedical Sciences (No. 1903131520-2). Animals were housed 3 per cage in a pathogen-free environment on a 12-h light cycle at 22 ± 2 °C with standard chow (CLEA Japan, Tokyo) and had free access to tap water.

### 4.7. Skeletal and Histological Analysis

Skeletal preparations were stained with alizarin red S and alcian blue, as described previously [4]. For light microscopy, we fixed tissues from embryos at E14.5, E16.5, and E18.5 in 4% paraformaldehyde/0.1 M phosphate buffer, embedded them in paraffin, and prepared 7-μm sections to perform H-E staining, safranin O staining, and TRAP staining. For the analysis of cell proliferation, we intra-peritoneally injected pregnant mice with 50 μg of BrdU/g of body weight 1 h before sacrifice. We processed the embryos for histological analysis and detected BrdU incorporation using a BrdU staining kit (Invitrogen, Carlsbad, CA, USA). Apoptosis was analyzed by TUNEL staining using the ApopTag peroxidase In Situ apoptosis detection kit (Sigma-Aldrich, St. Louis, MO, USA). The frequencies of BrdU- or TUNEL-positive cells were calculated using ImageJ software.

### 4.8. In Situ Hybridization

For in situ hybridization, single-stranded RNA probes labeled with digoxigenin-11-UTP were prepared using a DIG RNA labeling kit (Roche Biochemica) according to the manufacturer’s instructions. Sections were hybridized using mouse *Col2a1*, *Pthr1*, *Ihh*, *Col10a1*, *Mmp13*, *Ibsp*, *Spp1*, *Col1a1*, and *Bglap2* antisense probes, as described previously [6,37]. In situ hybridization using sense probes produced no significant signals. In situ hybridization was carried out as described previously [4], and the sections were counterstained with methyl green.

### 4.9. Microarray Analysis

RNA from the hind limbs of 5 wild-type embryos at E15.5 were mixed into one RNA sample, and RNA from two *Hck*^CA^ tg (tg1 and tg2) embryos were used individually in microarray analysis. Each sample was amplified, labeled, and hybridized using the Agilent SurePrint G3 mouse gene expression microarray 8 × 60 K (Agilent Technologies, Santa Clara, Calif., USA) according to the manufacturer’s instructions. Data were standardized using background correction and quantile normalization.

### 4.10. Heatmap, Hierarchical Clustering, and Functional Annotations

Raw expression values of wild-type and *Hck*^CA^ tg embryos were converted into log2 values and represented in a heatmap using Gene Cluster 3.0. To compare transcript levels between wild-type and *Hck*^CA^ tg samples, microarray probes were grouped into probe sets (gene), and the average signal intensity was log2-transformed and subjected to dendrogram and hierarchical clustering heat maps, with uncentered Pearson correlation and average linkage, using Gene Cluster 3.0. These data were visualized by Java Treeview. For functional analysis, modules of interest on significantly differentially expressed genes were further characterized by GO terms from all available domains (biological processes, molecular functions, and cellular components). Parameters comprising a cut-off false discovery rate (FDR) < 10^−4^ and enrichment of >2 were set in order to select a manageable number of significant genes. Enriched clusters of GO terms were further processed to remove redundancy based on semantic similarities with the SimRel algorithm using the REVIGO web tool.

### 4.11. Statistical Analysis

Values are shown as the mean ± SD. Statistical analyses were performed by the Student’s *t*-test. A *p*-value of less than 0.05 was considered significant.

## Supplementary Materials

Supplementary materials can be found at https://www.mdpi.com/1422-0067/21/8/2682/s1.
