# Supplementary material for: Expression of a Constitutively Active Form of Hck in Chondrocytes Activates Wnt and Hedgehog Signaling Pathways, and Induces Chondrocyte Proliferation in Mice
Source: Int J Mol Sci. 2020 Apr 12;21(8):2682. doi: 10.3390/ijms21082682 (PMC7215647; doi:10.3390/ijms21082682)
Supplement: Supplementary file 1 [file ijms-21-02682-s001.pdf]

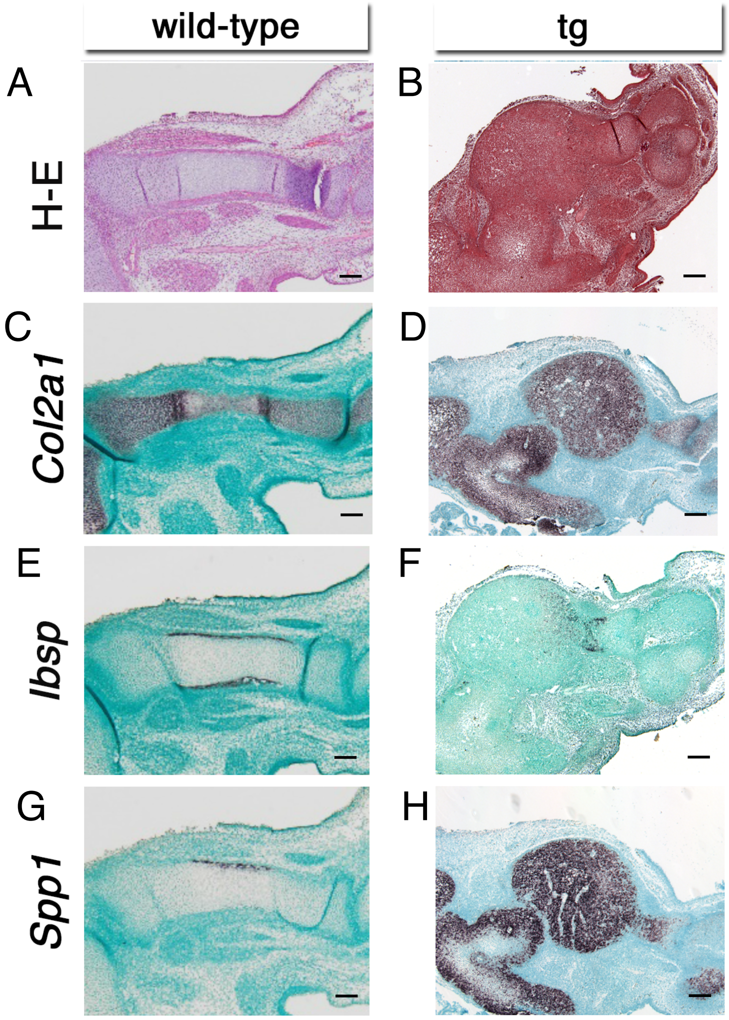

Supplementary Figure S1

Histological analyses of the tibia in *Hck*^{CA} tg embryo at E14.5 (A and B) H-E staining in wild-type (A) and *Hck*^{CA} tg (B) embryos. (C-H) In situ hybridization using *Col2a1* (C, D), *Ibsp* (E, F), and *Spp1* (G, H) probes in wild-type (C, E, G) and *Hck*^{CA} tg (D, F, H) embryos. Scale bars: 200 µm.

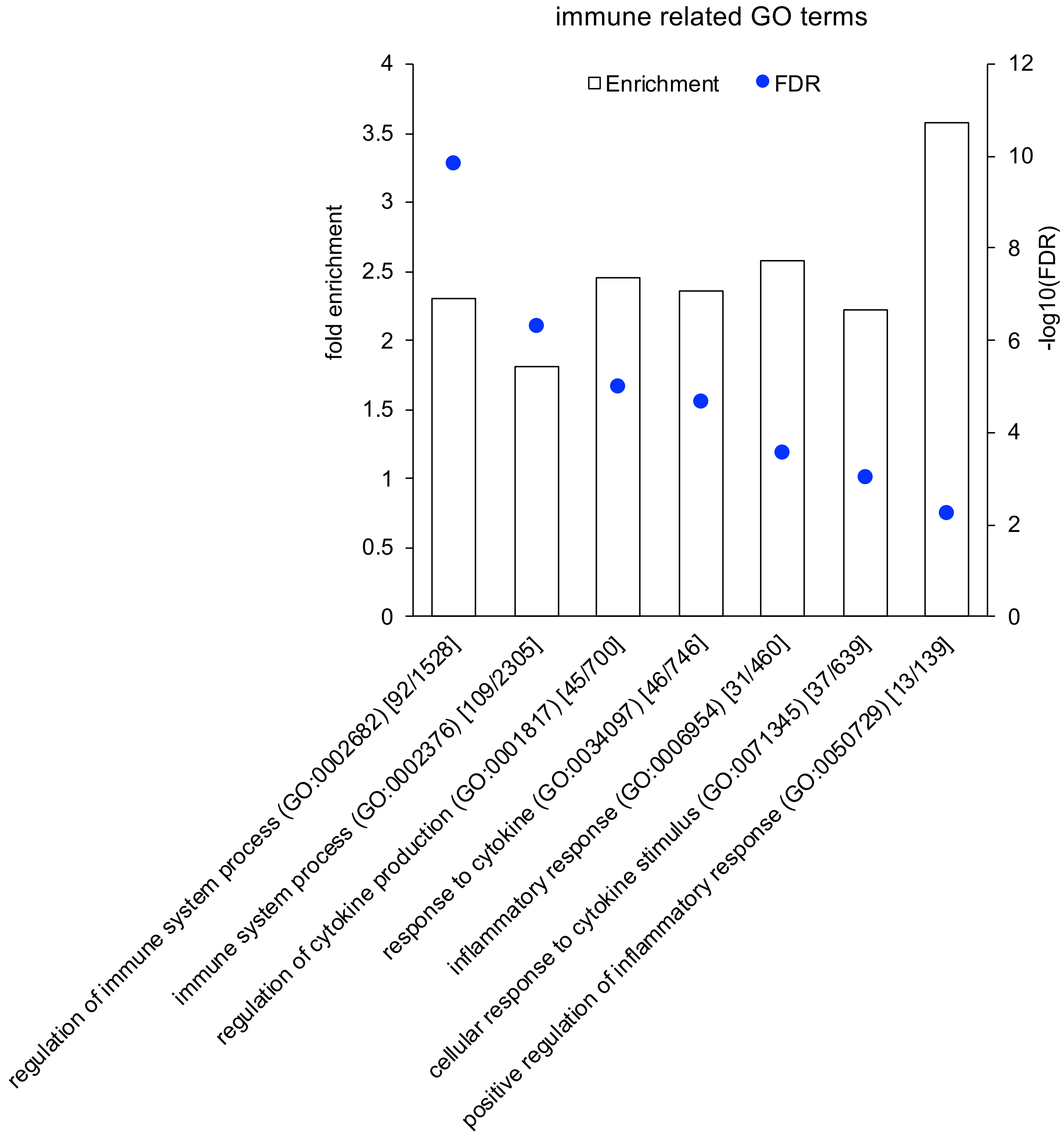

Supplementary Figure S2

Enriched GOs related to inflammation in both tg1 and tg2.

Supplementary Table S1

age	number of embryos	number of tg embryos
E14.5	71	16
E15.5	106	20
E16.5	61	19
E18.5	59	19

Supplementary Table S1
The production frequencies of *Hck*^{CA} tg embryos

Supplementary Table S2. Microarray data of the genes included in the top 20 GOs enrichment in Fig. 6D

Gene symbol	Accession #	Gene name	*Fold change*	
			tg1	tg2
Abca1	NM_013454	ATP-binding cassette, sub-family A	1.6201	2.3511
Acan	NM_007424	aggrecan	2.5036	2.6289
Ace	NM_009598	angiotensin I converting enzyme (peptidyl-dipeptidase A) 1	1.5411	4.0486
Acp5	NM_001102405	acid phosphatase 5, tartrate resistant	1.7879	1.8162
Adam12	NM_007400	a disintegrin and metallopeptidase domain 12 (meltrin alpha)	2.6263	2.1547
Adamts12	NM_175501	a disintegrin-like and metallopeptidase (reprolysin type) with thrombospondin type 1 motif, 12	2.1908	1.9663
Adamts18	NM_172466	a disintegrin-like and metallopeptidase (reprolysin type) with thrombospondin type 1 motif, 18	1.5703	2.4557
Adamts3	AK031900	RIKEN full-length enriched library, clone:6330442E02, similar to A DISINTEGRIN-LIKE AND METALLOPROTEASE DOMAIN WITH THROMBOSPONDIN TYPE I MOTIFS 3 (FRAGMENT) [Homo sapiens]	2.3368	2.8436
Adamts9	ENSMUST00000130314	a disintegrin-like and metallopeptidase (reprolysin type) with thrombospondin type 1 motif, 9	1.6763	1.7598
Adk	NM_001243041	adenosine kinase	1.6670	1.5702
Adm2	NM_182928	adrenomedullin 2	3.3864	2.9402
Aggf1	ENSMUST00000161671	angiogenic factor with G patch and FHA domains 1	1.8845	2.1761
Aire	NM_009646	autoimmune regulator (autoimmune polyendocrinopathy candidiasis ectodermal dystrophy)	11.2278	5.4691
Aldoc	BC004802	aldolase C, fructose-bisphosphate	4.5295	6.5444
Alpk1	NM_027808	alpha-kinase 1	2.3274	3.1132
Alpl	NM_007431	alkaline phosphatase	1.7184	3.0648
Als2	NM_028717	amyotrophic lateral sclerosis 2 (juvenile)	2.0823	2.2938
Ang3	NM_001123394	angiogenin, ribonuclease A family, member 3	2.1592	3.2554
Ank	NM_020332	progressive ankylosis	1.9642	4.6648
Ankrd6	NM_001012450	ankyrin repeat domain 6	2.0855	1.5585
Anxa1	AK037064	RIKEN full-length enriched library, clone:9930106N19	1.7007	2.5213
Anxa3	NM_013470	annexin A3	1.5536	3.4744
Anxa9	NM_023628	annexin A9	1.9438	1.5117
Apobec3	NM_001160415	apolipoprotein B mRNA editing enzyme, catalytic polypeptide 3	1.5731	4.5647
Apoc3	NM_001289756	apolipoprotein C-III	2.5385	2.1574
Arc	NM_018790	activity regulated cytoskeletal-associated protein	3.2708	3.6253
Arhgap4	NM_138630	Rho GTPase activating protein 4	1.7633	2.1929
Arid3b	ENSMUST00000171444	AT rich interactive domain 3B	1.5786	2.2601
Atp1b2	NM_013415	ATPase, Na+/K+ transporting, beta 2 polypeptide	3.4475	1.8631
Atp6v1c2	NM_133699	ATPase, H+ transporting, lysosomal V1 subunit C2	7.6120	9.9028
Avil	NM_009635	advillin	2.1479	2.4846
Bambi	NM_026505	BMP and activin membrane-bound inhibitor	2.0502	1.9195
Batf	NM_016767	basic leucine zipper transcription factor, ATF-like	1.8487	3.5479
Batf3	NM_030060	basic leucine zipper transcription factor, ATF-like 3	1.6181	2.0004
BC021891	NM_145608	cDNA sequence BC021891	2.6368	2.3953
Bcl3	NM_033601	B cell leukemia/lymphoma 3	1.5267	4.8416
Bend6	NM_177235	BEN domain containing 6	3.7260	5.0341
Bmp5	NM_007555	bone morphogenetic protein 5	1.6229	1.5189
Bmp7	NM_007557	bone morphogenetic protein 7	2.1634	2.6728
Bnip3	NM_009760	BCL2/adenovirus E1B interacting protein 3	1.9322	2.0284
Bpifb1	NM_001012392	BPI fold containing family B, member 1	9.0605	20.0784
Btla	NM_001037719	B and T lymphocyte associated	3.3116	5.1500
C2cd4a	NM_001163143	C2 calcium-dependent domain containing 4A	1.9554	1.9713
Car2	NM_009801	carbonic anhydrase 2	3.2164	2.1371
Car9	NM_139305	carbonic anhydrase 9	5.1706	5.0170
Casp4	NM_007609	caspase 4, apoptosis-related cysteine peptidase	1.9681	8.6514
Casp9	NM_015733	caspase 9	1.6858	1.6003
Cd200	NM_010818	CD200 antigen	1.5989	2.9491
Cd2ap	NM_009847	CD2-associated protein	1.5136	1.9932
Cd3e	NM_007648	CD3 antigen, epsilon polypeptide	2.2736	3.3955
Cd84	NM_013489	CD84 antigen	1.7986	1.9799
Cd9	NM_007657	CD9 antigen	1.5884	1.8946
Cdc42se1	NM_172395	CDC42 small effector 1	1.5387	2.3090
Cdh10	NM_009865	cadherin 10	1.5123	2.9866
Cdh2	ENSMUST00000152779	cadherin 2	1.6909	1.8391
Cdk5rap1	NM_025876	CDK5 regulatory subunit associated protein 1	2.7163	3.3500
Cdkn2a	NM_009877	cyclin-dependent kinase inhibitor 2A	2.3779	25.0791
Cdkn2b	NM_007670	cyclin-dependent kinase inhibitor 2B	2.1048	10.7555
Celf4	NM_001146295	CUGBP, Elav-like family member 4	1.6580	1.8557
Cemip	NM_030728	cell migration inducing protein, hyaluronan binding	1.7199	2.9715
Cep250	NM_001129999	centrosomal protein 250	1.5013	1.6362
Cfh	AK075670	RIKEN full-length enriched library, clone:1110061K10, complement component factor h,	1.5616	1.5236
Cfp	NM_008823	complement factor properdin	1.6748	2.1444
Chac1	NM_026929	ChaC, cation transport regulator 1	1.9825	2.6040
Chad	NM_007689	chondroadherin	1.9393	3.2238
Chst11	NM_021439	carbohydrate sulfotransferase 11	2.1058	2.0148
Clec12a	NM_177686	C-type lectin domain family 12, member a	1.6992	1.7515
Clic4	ENSMUST00000143370	chloride intracellular channel 4 (mitochondrial)	1.8854	2.0387
Cntnap1	NM_016782	contactin associated protein-like 1	1.5076	1.7548
Cntrl	NM_012018	centriolin	1.5271	1.7492
Col11a2	NM_009926	collagen, type XI, alpha 2	2.6478	2.3100
Col24a1	NM_027770	collagen, type XXIV, alpha 1	1.8484	1.5684
Col27a1	ENSMUST00000125504	collagen, type XXVII, alpha 1	2.3956	2.7658
Col2a1	NM_031163	collagen, type II, alpha 1	2.7634	3.0426
Col9a1	NM_007740	collagen, type IX, alpha 1	2.9390	1.8291
Col9a3	NM_009936	collagen, type IX, alpha 3	2.5262	2.8528
Comp	NM_016685	cartilage oligomeric matrix protein	2.3275	3.4648
Cpe	NM_013494	carboxypeptidase E	1.8845	1.5882
Cpne1	NM_170588	copine I	2.1075	2.0495
Cspg4	NM_139001	chondroitin sulfate proteoglycan 4	2.0789	1.9601
Ctgf	NM_010217	connective tissue growth factor	1.6524	1.7125
Ctla4	NM_009843	cytotoxic T-lymphocyte-associated protein 4	6.6324	15.0247
Cubn	NM_001081084	cubilin	2.3452	1.7024
Cyth4	AK036679	RIKEN full-length enriched library, clone:9830160N02, egional homology to PLECKSTRIN, SEC7 AND COILED/COIL DOMAINS 4 homolog [Homo sapiens]	2.0638	2.4117
Cytip	NM_139200	cytohesin 1 interacting protein	5.8842	6.1389
Daam2	NM_001008231	dishevelled associated activator of morphogenesis 2	2.4345	2.9696
Dapk2	ENSMUST00000129442	death-associated protein kinase 2	2.0769	3.7546
Ddx41	NM_134059	DEAD (Asp-Glu-Ala-Asp) box polypeptide 41	1.7258	2.1971
Dhh	NM_007857	desert hedgehog	1.6240	3.3593
Dixdc1	ENSMUST00000149717	DIX domain containing 1	1.6152	1.7459
Dkk1	NM_010051	dickkopf homolog 1 (Xenopus laevis)	1.5261	3.4158
Dlx2	NM_010054	distal-less homeobox 2	2.5431	1.6403
Dlx4	NM_007867	distal-less homeobox 4	2.7402	4.0737
Dlx6	NM_010057	distal-less homeobox 6	1.5441	1.8324
Dnase1l2	NM_025718	deoxyribonuclease 1-like 2	1.5357	1.8523
Dnm3	NM_001038619	dynamin 3	2.2875	1.5452
Dusp15	NM_001159376	dual specificity phosphatase-like 15	6.8273	2.0822
Dusp5	ENSMUST00000038287	dual specificity phosphatase 5	1.5078	3.1362
Edil3	NM_010103	EGF-like repeats and discoidin I-like domains 3	1.7825	2.0728
Egr3	NM_001289925	early growth response 3	8.2988	8.0835

Supplementary Table S2 (cont.). Microarray data of the genes included in the top 20 GOs enrichment in Fig. 6D

Gene symbol	Accession #	Gene name	*Fold change*	
			tg1	tg2
Ehd3	NM_020578	EH-domain containing 3	2.1578	2.2147
Eif4g3	AK137712	RIKEN full-length enriched library, clone:9930106D06, eukaryotic translation initiation factor 4 gamma, 3	1.6843	1.9396
Ell2	NM_138953	elongation factor RNA polymerase II 2	1.6630	2.5589
Emb	NM_010330	embigin	2.9817	6.3742
Eno1	NM_023119	enolase 1, alpha non-neuron	1.6655	1.6504
Enpp1	NM_008813	ectonucleotide pyrophosphatase/phosphodiesterase 1	2.0527	2.1301
Eps8	ENSMUST00000139753	epidermal growth factor receptor pathway substrate 8	2.2542	2.4595
Etv4	NM_008815	ets variant 4	1.8318	1.5291
Fabp4	NM_024406	fatty acid binding protein 4, adipocyte	1.5754	2.8087
Fbxl2	NM_178624	F-box and leucine-rich repeat protein 2	1.6714	1.5195
Fgd2	NM_013710	FYVE, RhoGEF and PH domain containing 2	1.6055	1.5292
Fgd3	NM_015759	FYVE, RhoGEF and PH domain containing 3	3.1855	2.8481
Fgf3	ENSMUST00000105898	fibroblast growth factor 3	1.5654	2.0184
Fgfr3	NM_001163215	fibroblast growth factor receptor 3	2.7910	3.0377
Fgfrl1	NM_054071	fibroblast growth factor receptor-like 1	1.5023	1.9093
Flt1	ENSMUST00000110529	FMS-like tyrosine kinase 1	1.8451	1.6570
Fn1	NM_001276413	fibronectin 1	2.4438	2.6556
Foxa2	NM_001291067	forkhead box A2	3.3275	2.2034
Foxa3	NM_008260	forkhead box A3	3.2899	3.0582
Fry1	ENSMUST00000153923	furry homolog-like (Drosophila)	1.6771	2.8739
Frzb	NM_011356	frizzled-related protein	1.8255	3.7149
Fyb	NM_001278269	FYN binding protein	1.6170	5.9774
Fzd9	NM_010246	frizzled homolog 9 (Drosophila)	1.6001	1.9398
Gabra5	NM_176942	gamma-aminobutyric acid	1.6058	4.6497
Gal3st1	NM_016922	galactose-3-O-sulfotransferase 1	2.2692	1.5312
Gif	NM_008118	gastric intrinsic factor	1.8954	2.3709
Gja1	XM_011243119	gap junction protein, alpha 1	2.2711	1.5612
Gja8	NM_008123	gap junction protein, alpha 8	21.3889	6.6928
Gm11127	NM_001199967	predicted gene 11127	2.0312	5.8561
Gm13275	NM_001085533	predicted gene 13275	1.6017	2.4412
Gper1	NM_029771	G protein-coupled estrogen receptor 1	1.8227	3.1705
Gpr68	NM_175493	G protein-coupled receptor 68	3.5793	8.2758
Gucy2d	NM_001130693	guanylate cyclase 2d	1.5785	1.6132
H2-Bl	XM_011238153	histocompatibility 2, blastocyst	2.5742	4.7521
H2-K1	NM_001001892	histocompatibility 2, K1, K region	1.5590	6.0320
H2-L	NM_001267808	histocompatibility 2, D region locus L	1.5186	5.3589
H2-Q2	NM_010392	histocompatibility 2, Q region locus 2	1.8825	5.3471
H2-Q8	NM_023124	histocompatibility 2, Q region locus 8	1.8041	5.1148
H2-T23	NM_010398	histocompatibility 2, T region locus 23	1.6700	6.5374
Hapln1	NM_013500	hyaluronan and proteoglycan link protein 1	2.4504	1.8927
Hck	NM_001172117	hemopoietic cell kinase	1.6163	4.2059
Hcst	NM_011827	hematopoietic cell signal transducer	2.2545	3.7062
Hdac9	NM_024124	histone deacetylase 9	2.2139	3.1437
Hecw2	NM_172655	HECT, C2 and WW domain containing E3 ubiquitin protein ligase 2	1.5980	1.7722
Hey1	NM_010423	hairy/enhancer-of-split related with YRPW motif 1	1.5740	1.5697
Hip1r	NM_145070	huntingtin interacting protein 1 related	2.5220	2.6437
Hivep3	ENSMUST00000106307	human immunodeficiency virus type I enhancer binding protein 3	2.9746	3.1843
Hpgd	NM_008278	hydroxyprostaglandin dehydrogenase 15	1.5034	2.1432
Hpn	NM_008281	hepsin	1.5402	2.6339
Id1	NM_010495	inhibitor of DNA binding 1	1.9581	1.5815
Ifitm1	NM_001112715	interferon induced transmembrane protein 1	1.9323	5.5506
Ifitm5	NM_053088	interferon induced transmembrane protein 5	2.2298	1.9802
Ihh	ENSMUST00000189364	Indian hedgehog	3.2654	5.6386
Il12rb1	NM_008353	interleukin 12 receptor, beta 1	4.0324	10.5374
Il17ra	NM_008359	interleukin 17 receptor A	1.8363	1.7790
Il18r1	NM_008365	interleukin 18 receptor 1	8.7951	12.7355
Iqgap1	AK163056	RIKEN full-length enriched library, clone:A430107C16, IQ motif containing GTPase activating protein 1	2.1734	2.9945
Iqgap2	NM_027711	IQ motif containing GTPase activating protein 2	1.5508	1.9524
Iqsec1	NM_001134383	IQ motif and Sec7 domain 1	2.5131	2.6492
Irf1	NM_008390	interferon regulatory factor 1	1.8450	3.7933
Itga3	NM_013565	integrin alpha 3	1.5466	1.8845
Itga6	NM_008397	integrin alpha 6	1.8872	2.3791
Itgal	NM_008400	integrin alpha L	1.6275	2.7839
Itgb2	NM_008404	integrin beta 2	3.5450	2.4947
Itgb3	NM_016780	integrin beta 3	1.6454	5.2002
Jak3	NM_010589	Janus kinase 3	2.3181	4.6818
Jph4	NM_177049	junctophilin 4	1.8159	2.3653
Kcnq1	FJ425887	potassium voltage-gated channel subfamily Q member 1 variant 4	2.8832	3.5102
Kctd16	NM_026135	potassium channel tetramerisation domain containing 16	1.8457	1.6666
Kdm7a	NM_001033430	lysine (K)-specific demethylase 7A	2.2222	2.0958
Klf15	NM_023184	Kruppel-like factor 15	1.9363	1.6434
Klk8	NM_008940	kallikrein related-peptidase 8	1.7642	2.1758
Lat	NM_010689	linker for activation of T cells	1.5633	3.0780
Lbh	NM_029999	limb-bud and heart	3.4767	3.6667
Lclat1	NM_001081071	lysocardiolipin acyltransferase 1	1.5489	1.5477
Lcp1	NM_008879	lymphocyte cytosolic protein 1	1.5686	2.1851
Ldlr	AK161104	RIKEN full-length enriched library, clone:4732431M03, low density lipoprotein receptor	1.7895	2.3117
Lef1	NM_010703	lymphoid enhancer binding factor 1	5.3778	3.9223
Lepr	ENSMUST00000128948	leptin receptor	1.6815	1.6553
Lgals3	NM_001145953	lectin, galactose binding, soluble 3	1.8753	4.1884
Litaf	NM_019980	LPS-induced TN factor	1.5597	4.0393
Lmntd2	NM_028050	lamin tail domain containing 2	2.3097	2.2155
Lrp4	NM_172668	low density lipoprotein receptor-related protein 4	1.6506	2.2453
Lrp5	AK081025	RIKEN full-length enriched library, clone:B930069L06, low density lipoprotein receptor-related protein 5	1.5220	1.6203
Lrrc8c	NM_133897	leucine rich repeat containing 8 family, member C	1.6151	1.7281
Lsr	NM_017405	lipolysis stimulated lipoprotein receptor	1.8544	2.3271
Luzp1	NM_024452	leucine zipper protein 1	2.2723	2.5162
M1ap	NM_033079	meiosis 1 associated protein	3.4742	8.1578
Map3k5	NM_008580	mitogen-activated protein kinase kinase kinase 5	1.6519	1.6732
Matn3	NM_010770	matrilin 3	4.5228	3.0554
Matn4	NM_013592	matrilin 4	2.5349	1.9939
Mcf2l	NM_001159486	mcf.2 transforming sequence-like	4.2411	16.6233
Mcoln2	NM_026656	mucolipin 2	3.1364	2.1144
Me2	AK080403	RIKEN full-length enriched library, clone:A630098G22, similar to NAD-DEPENDENT MALIC ENZYME, MITOCHONDRIAL PRECURSOR (EC 1.1.1.38) (NAD-ME) [Homo sapiens]	1.6286	1.6249
Mef2c	ENSMUST00000196832	ncer factor 2C	1.5611	2.0814
Mfn2	AK049583	RIKEN full-length enriched library, clone:C430046A10, similar to HYPERTENSION RELATED PROTEIN 1 [Mus musculus]	1.6205	1.6959
Mgarp	NM_026358	mitochondria localized glutamic acid rich protein	2.0571	2.5904
Mlph	NM_053015	melanophilin	2.3309	4.8934
Mlx	NM_011550	MAX-like protein X	1.5254	2.1381
Mmp9	NM_013599	matrix metallopeptidase 9	2.7573	2.4887

Supplementary Table S2 (cont.). Microarray data of the genes included in the top 20 GOs enrichment in Fig. 6D

Gene symbol	Accession #	Gene name	*Fold change*	
			tg1	tg2
Mpv17l	NM_033564	Mpv17 transgene, kidney disease mutant-like	1.5079	1.9143
Msn	ENSMUST00000149985	moesin	1.5099	1.5349
Mthfd1l	NM_001170786	methylenetetrahydrofolate dehydrogenase (NADP+ dependent) 1-like	1.5959	2.1780
Myd88	NM_010851	myeloid differentiation primary response gene 88	1.5404	4.4257
Myo1e	NM_181072	myosin IE	1.6896	2.5381
Nab2	NM_001122895	Ngfi-A binding protein 2	1.6405	1.8738
Ncf2	NM_010877	neutrophil cytosolic factor 2	3.9447	2.9417
Ncmap	NM_001243306	noncompact myelin associated protein	3.3478	5.2611
Nfkbiz	NM_030612	nuclear factor of kappa light polypeptide gene enhancer in B cells inhibitor, zeta	1.9690	3.3608
Nr4a2	NM_013613	nuclear receptor subfamily 4, group A, member 2	1.8399	2.6237
Nrgn	NM_022029	neurogranin	1.9136	2.3020
Nrn1	NM_153529	neuritin 1	1.8306	1.9732
Ocm	NM_033039	oncomodulin	2.6417	6.4156
Ocstamp	NM_029021	osteoclast stimulatory transmembrane protein	1.5172	1.6163
Oog2	NM_198661	oogenesin 2	1.8371	2.4998
Osmr	NM_011019	oncostatin M receptor	1.7709	4.0955
Pak3	NM_008778	p21 protein	2.0496	2.7061
Panx3	NM_172454	pannexin 3	2.4537	2.8327
Parvg	NM_001162500	parvin, gamma	2.4955	2.0608
Pawr	NM_054056	PRKC, apoptosis, WT1, regulator	1.7485	1.7097
Pcp4	NM_008791	Purkinje cell protein 4	2.2863	1.7668
Pcsk6	NM_011048	proprotein convertase subtilisin/kexin type 6	1.9690	1.7974
Pde4dip	NM_001289701	phosphodiesterase 4D interacting protein	1.9413	2.2364
Pdgfb	AK087840	RIKEN full-length enriched library,<br>clone:E330027M19, platelet derived growth factor, B polypeptide	1.5371	1.6858
Peli1	NM_023324	pellino 1	1.5161	1.5350
Pfkl	NM_008826	phosphofructokinase, liver, B-type	1.9649	1.9640
Phc3	NM_153421	polyhomeotic-like 3	1.5703	1.7901
Phex	NM_011077	phosphate regulating endopeptidase homolog, X-linked	1.6895	2.4985
Phospho1	NM_153104	phosphatase, orphan 1	1.5720	2.8458
Pik3ip1	ENSMUST00000139139	phosphoinositide-3-kinase interacting protein 1	1.5568	1.8516
Pik3r1	NM_001077495	phosphatidylinositol 3-kinase, regulatory subunit, polypeptide 1	1.6276	1.8801
Pikfyve	NM_011086	phosphoinositide kinase, FYVE finger containing	2.0337	1.9931
Pla2g7	NM_013737	phospholipase A2, group VII	2.3110	4.6145
Plaur	NM_011113	plasminogen activator, urokinase receptor	1.5019	5.0671
Plb1	NM_001081407	phospholipase B1	1.5676	2.1463
Plcb1	NM_019677	phospholipase C, beta 1	1.7202	1.6712
Plcg2	NM_172285	phospholipase C, gamma 2	1.5379	1.7218
Plekha4	NM_148927	pleckstrin homology domain containing,	1.5961	1.7499
Plekhg6	NM_198604	pleckstrin homology domain containing,	1.8748	2.4629
Plod3	NM_011962	procollagen-lysine, 2-oxoglutarate 5-dioxygenase 3	1.5401	2.2122
Plscr1	NM_011636	phospholipid scramblase 1	1.7528	4.2975
Pltp	NM_011125	phospholipid transfer protein	1.9162	5.8924
Plxnb1	NM_172775	plexin B1	2.1860	1.7591
Pof1b	NM_181579	premature ovarian failure 1B	2.4596	1.6478
Pofut1	NM_080463	protein O-fucosyltransferase 1	2.1961	1.8352
Ppm1e	NM_177167	protein phosphatase 1E	1.8789	2.4128
Ppm1h	NM_001110218	protein phosphatase 1H	1.7814	1.7590
Prelp	NM_054077	proline arginine-rich end leucine-rich repeat	2.5651	1.9895
Prex2	NM_029525	phosphatidylinositol-3,4,5-trisphosphate-dependent Rac exchange factor 2	3.1938	1.5074
Prkch	NM_008856	protein kinase C, eta	2.0907	1.6390
Prl2c1	NM_001045532	Prolactin family 2, subfamily c, member 1	1.6293	5.0764
Prr15	NM_030024	proline rich 15	3.1808	3.8972
Prr5l	ENSMUST00000127358	proline rich 5 like	2.1321	3.8327
Prss56	NM_027084	protease, serine 56	1.6675	2.2829
Ptch1	NM_008957	patched homolog 1	2.2839	1.9875
Ptger4	NM_008965	prostaglandin E receptor 4	1.5633	2.3569
Ptgs2	NM_011198	prostaglandin-endoperoxide synthase 2	2.1229	12.4992
Pth1r	NM_011199	parathyroid hormone 1 receptor	1.6736	2.7450
Ptk2b	NM_001162365	PTK2 protein tyrosine kinase 2 beta	2.1684	4.0562
Ptprc	ENSMUST00000185570	protein tyrosine phosphatase, receptor type, C	2.7270	6.4030
Ptpre	NM_011212	protein tyrosine phosphatase, receptor type, E	1.5024	1.5717
Ptprj	NM_001135657	protein tyrosine phosphatase, receptor type, J	1.7486	4.6399
Rab11fip4	NM_175543	RAB11 family interacting protein 4	1.6527	1.8516
Rabl2	NM_026817	RAB, member RAS oncogene family-like 2	1.5653	1.8041
Rbpjl	NM_009036	recombination signal binding protein for immunoglobulin kappa J region-like	3.9121	2.6759
Recql5	ENSMUST00000144824	RecQ protein-like 5	1.5045	2.7832
Rffl	NM_026097	ring finger and FYVE like domain containing protein	1.8910	1.7481
Ripk2	NM_138952	receptor	1.9423	3.4161
Runx1	NM_009821	runt related transcription factor 1	2.6409	4.3748
Runx2	NM_009820	runt related transcription factor 2	2.3827	3.0484
Runx3	NM_019732	runt related transcription factor 3	3.0934	2.0285
S100a1	NM_011309	S100 calcium binding protein A1	2.2930	2.6241
S100a4	NM_011311	S100 calcium binding protein A4	2.0571	3.8500
Samd4b	NM_175021	sterile alpha motif domain containing 4B	1.7270	1.6687
Satb2	NM_139146	special AT-rich sequence binding protein 2	1.5211	2.3673
Sbno2	BC064113	strawberry notch homolog 2	1.7381	6.2630
Scd1	NM_009127	stearoyl-Coenzyme A desaturase 1	1.6057	2.1629
Scin	NM_009132	scinderin	2.4570	2.6279
Sdcbp	ENSMUST00000155247	syndecan binding protein	2.0106	1.5387
Sema7a	NM_011352	sema domain, immunoglobulin domain (Ig), and GPI membrane anchor,<br>(semaphorin) 7A	2.4327	4.1282
Sept5	NM_213614	septin 5	2.0341	1.8204
Serinc5	AK163675	RIKEN full-length enriched library, clone:B930007O12, hypothetical TMS membrane protein/tumour differentially expressed protein/Cysteine-rich region profile/2Fe-2S ferredoxin, iron-sulfur binding site containing protein	2.1731	3.8011
Serpinb6b	NM_011454	serine (or cysteine) peptidase inhibitor, clade B, member 6b	1.5747	2.2138
Serpine2	ENSMUST00000191026	serine (or cysteine) peptidase inhibitor, clade E, member 2	1.9040	4.5260
Slc1a1	NM_009199	solute carrier family 1 (neuronal/epithelial high affinity glutamate transporter, system Xag), member 1	6.6874	9.6248
Slc1a3	NM_148938	solute carrier family 1 (glial high affinity glutamate transporter), member 3	2.3815	2.1883
Slc26a2	NM_007885	solute carrier family 26 (sulfate transporter), member 2	1.8116	2.5517
Slc35d1	NM_177732	solute carrier family 35 (UDP-glucuronic acid/UDP-N-acetylgalactosamine dual transporter), member D1	1.8713	1.7243
Slc36a2	NM_153170	solute carrier family 36 (proton/amino acid symporter), member 2	1.5907	2.3405
Slc38a3	NM_023805	solute carrier family 38, member 3	2.2417	2.0720
Smpd3	NM_021491	sphingomyelin phosphodiesterase 3, neutral	1.7497	3.3005
Snx10	NM_028035	sorting nexin 10	1.9266	1.8202
Sorbs1	NM_178362	sorbin and SH3 domain containing 1	1.9340	1.5388
Sort1	NM_001271599	sortilin 1	1.5781	1.6217
Sox5	ENSMUST00000129050	SRY (sex determining region Y)-box 5	2.0441	1.6107
Sox6	NM_011445	SRY (sex determining region Y)-box 6	2.1130	2.0565
Sox9	NM_011448	SRY (sex determining region Y)-box 9	2.8803	2.1901
Sp7	NM_130458	Sp7 transcription factor 7	2.2511	3.9924
Spink6	NM_001013797	serine peptidase inhibitor, Kazal type 6	9.7335	10.4381
Spp1	NM_009263	secreted phosphoprotein 1	3.3440	10.0947

Supplementary Table S2 (cont.). Microarray data of the genes included in the top 20 GOs enrichment in Fig. 6D

Gene symbol	Accession #	Gene name	*Fold change*	
			tg1	tg2
Ssh2	ENSMUST00000180394	slingshot homolog 2 (Drosophila)	1.5231	1.6784
Sspo	NM_173428	SCO-spondin	1.6423	2.4930
Ssu2	ENSMUST00000143134	ssu-2 homolog (C. elegans)	1.9176	2.5805
St14	NM_011176	suppression of tumorigenicity 14 (colon carcinoma)	4.0621	6.3998
St5	NM_001001326	suppression of tumorigenicity 5	2.2071	1.7137
Stat4	NM_011487	signal transducer and activator of transcription 4	1.8237	2.5008
Stc2	NM_011491	stanniocalcin 2	3.2699	1.8859
Synpo	ENSMUST00000137894	synaptopodin	1.7703	1.6703
Syt8	NM_018802	synaptotagmin VIII	2.9375	5.6153
Tanc1	ENSMUST00000142573	tetratricopeptide repeat, ankyrin repeat and coiled-coil containing 1	1.9317	1.6475
Tcf7	ENSMUST00000086844	transcription factor 7, T cell specific	1.5152	2.0899
Tcirg1	NM_016921	T cell, immune regulator 1, ATPase, H+ transporting, lysosomal V0 protein A3	1.5052	1.6404
Tenm1	ENSMUST00000126161	teneurin transmembrane protein 1	2.3128	1.7167
Tfeb	NM_001161722	transcription factor EB	2.3501	6.9477
Tfec	NM_031198	transcription factor EC	1.8248	2.9408
Tfrc	NM_011638	transferrin receptor	2.0221	1.9583
Tgfb1	NM_011577	transforming growth factor, beta 1	1.9206	3.9865
Tifa	NM_145133	TRAF-interacting protein with forkhead-associated domain	1.6110	3.2450
Timp1	NM_001044384	tissue inhibitor of metalloproteinase 1	2.8625	5.4598
Tmbim4	ENSMUST00000145015	transmembrane BAX inhibitor motif containing 4	2.4137	2.1482
Tmem173	NM_028261	transmembrane protein 173	1.8083	4.6743
Tmie	NM_146260	transmembrane inner ear	1.8893	4.3544
Tnfrsf13b	NM_021349	tumor necrosis factor receptor superfamily, member 13b	1.5137	1.6744
Tnfrsf1b	NM_011610	tumor necrosis factor receptor superfamily, member 1b	1.5727	5.8719
Tnfsf9	NM_009404	tumor necrosis factor (ligand) superfamily, member 9	1.7874	2.5648
Tns2	NM_153533	tensin 2	1.8746	1.8071
Tns3	ENSMUST00000134823	tensin 3	2.6885	2.1537
Trib3	NM_175093	tribbles homolog 3 (Drosophila)	3.5541	8.0583
Trpv4	NM_022017	transient receptor potential cation channel, subfamily V, member 4	2.3911	3.7284
Tssk6	NM_032004	testis-specific serine kinase 6	1.5440	1.7232
Ttc7	AK004107	RIKEN full-length enriched library, clone:1110035E02, hypothetical protein	1.9548	3.1583
Tyrobp	NM_011662	TYRO protein tyrosine kinase binding protein	1.6386	2.4356
Unc13a	ENSMUST00000175780	unc-13 homolog A (C. elegans)	2.2765	6.7653
Unc5c	NM_009472	unc-5 homolog C (C. elegans)	2.1780	1.6573
Vdr	AK156789	RIKEN full-length enriched library, clone:F830050A09, vitamin D receptor	1.8704	3.8818
Vegfa	NM_001025250	vascular endothelial growth factor A	3.0173	2.3642
Vldlr	NM_013703	very low density lipoprotein receptor	1.8485	2.1417
Vwa1	AK035229	RIKEN full-length enriched library, clone:9530003H10, inferred: Mus musculus von Willebrand factor A-related protein (Warp) mRNA, complete cds	1.6224	2.1281
Wwc2	AK036263	RIKEN full-length enriched library, clone:9630050D06	2.2922	2.6203
Wwp2	NM_025830	WW domain containing E3 ubiquitin protein ligase 2	2.0521	1.7784
Zc3hav1	NM_028864	zinc finger CCCH type, antiviral 1	1.6748	4.3624
Zeb2	BC050145	zinc finger homeobox 1b, mRNA	1.5068	1.6677
Zfp64	NM_009564	zinc finger protein 64	1.7883	2.0163
Zmiz1	ENSMUST00000159766	zinc finger, MIZ-type containing 1	1.8225	1.5070
Zscan10	NM_001033425	zinc finger and SCAN domain containing 10	1.6383	3.4450

Supplementary Table S3. Microarray data of the genes included in the enriched GOs related to inflammation in Supplementary Fig. 2

Gene symbol	Accession #	Gene name	*Fold change*	
			tg1	tg2
Ace	NM_009598	angiotensin I converting enzyme (peptidyl-dipeptidase A) 1	1.5411	4.0486
Acp5	NM_001102405	acid phosphatase 5, tartrate resistant (Acp5)	1.7879	1.8162
Adamts12	NM_175501	disintegrin-like and metallopeptidase (reprolysin type) with thrombospondin type 1 motif, 12	2.1908	1.9663
Adk	NM_001243041	adenosine kinase (Adk)	1.6670	1.5702
Aire	NM_009646	autoimmune regulator (autoimmune polyendocrinopathy candidiasis ectodermal dystrophy)	11.2278	5.4691
Alpk1	NM_027808	alpha-kinase 1	2.3274	3.1132
Alpl	NM_007431	alkaline phosphatase	1.7184	3.0648
Anxa1	AK037064	adult female vagina cDNA	1.7007	2.5213
Anxa3	NM_013470	annexin A3	1.5536	3.4744
Anxa9	NM_023628	annexin A9	1.9438	1.5117
Apobec3	NM_001160415	apolipoprotein B mRNA editing enzyme, catalytic polypeptide 3	1.5731	4.5647
Apod	NM_007470	apolipoprotein D	2.0123	5.1438
Batf	NM_016767	basic leucine zipper transcription factor, ATF-like	1.8487	3.5479
Batf3	NM_030060	basic leucine zipper transcription factor, ATF-like 3	1.6181	2.0004
Bcl3	NM_033601	B cell leukemia/lymphoma 3	1.5267	4.8416
Bnip3	NM_009760	BCL2/adenovirus E1B interacting protein 3	1.9322	2.0284
Bpifb1	NM_001012392	BPI fold containing family B, member 1	9.0605	20.0784
Btla	NM_001037719	B and T lymphocyte associated	3.3116	5.1500
C2cd4a	NM_001163143	C2 calcium-dependent domain containing 4A	1.9554	1.9713
Capg	NM_001042534	capping protein (actin filament), gelsolin-like	1.7965	3.1430
Capg	NM_001042534	capping protein (actin filament), gelsolin-like	1.7965	3.1430
Car2	NM_009801	carbonic anhydrase 2	3.2164	2.1371
Casp4	NM_007609	caspase 4, apoptosis-related cysteine peptidase	1.9681	8.6514
Ccrl2	NM_017466	chemokine (C-C motif) receptor-like 2	1.6160	4.8024
Cd200	NM_010818	CD200 antigen	1.5989	2.9491
Cd2ap	NM_009847	CD2-associated protein	1.5136	1.9932
Cd3e	NM_007648	CD3 antigen, epsilon polypeptide	2.2736	3.3955
Cd84	NM_013489	CD84 antigen	1.7986	1.9799
Cd9	NM_007657	CD9 antigen	1.5884	1.8946
Cdkn2a	NM_009877	cyclin-dependent kinase inhibitor 2A	2.3779	25.0791
Cdkn2b	NM_007670	cyclin-dependent kinase inhibitor 2B	2.1048	10.7555
Cfh	AK075670	RIKEN full-length enriched library, clone:1110061K10, complement component factor h	1.5616	1.5236
Cfp	NM_008823	complement factor properdin	1.6748	2.1444
Clec12a	NM_177686	C-type lectin domain family 12, member	1.6992	1.7515
Col24a1	NM_027770	collagen, type XXIV, alpha 1	1.8484	1.5684
Cspg4	NM_139001	chondroitin sulfate proteoglycan 4	2.0789	1.9601
Ctla4	NM_009843	cytotoxic T-lymphocyte-associated protein 4	6.6324	15.0247
Dapk2	ENSMUST00000129442	ated protein kinase 2	2.0769	3.7546
Dclre1c	NM_175683	DNA cross-link repair 1C, PSO2 homolog (S. cerevisiae)	1.6760	1.7291
Ddx41	NM_134059	DEAD (Asp-Glu-Ala-Asp) box polypeptide 41	1.7258	2.1971
Egr3	NM_001289925	early growth response 3	8.2988	8.0835
Eno1	NM_023119	enolase 1, alpha non-neuron	1.6655	1.6504
Enpp1	NM_008813	ectonucleotide pyrophosphatase/phosphodiesterase 1	2.0527	2.1301
Eps8	ENSMUST00000139753	epidermal growth factor receptor pathway substrate 8	2.2542	2.4595
Fabp4	NM_024406	fatty acid binding protein 4, adipocyte	1.5754	2.8087
Fgf3	ENSMUST00000105898	fibroblast growth factor 3	1.5654	2.0184
Fgfr3	NM_001163215	fibroblast growth factor receptor 3	2.7910	3.0377
Flt1	ENSMUST00000110529	FMS-like tyrosine kinase 1	1.8451	1.6570
Fn1	NM_001276413	fibronectin 1	2.4438	2.6556
Fyb	NM_001278269	FYN binding protein	1.6170	5.9774
Fzd9	NM_010246	frizzled homolog 9 (Drosophila)	1.6001	1.9398
Gif	NM_008118	gastric intrinsic factor	1.8954	2.3709
Gja1	XM_011243119	gap junction protein, alpha 1	2.2711	1.5612
Gm11127	NM_001199967	predicted gene 11127	2.0312	5.8561
Gm13275	NM_001085533	predicted gene 13275	1.6017	2.4412
Gper1	NM_029771	G protein-coupled estrogen receptor 1	1.8227	3.1705
Gpr68	NM_175493	G protein-coupled receptor 68	3.5793	8.2758
H2-Bl	XM_011238153	histocompatibility 2, blastocyst	2.5742	4.7521
H2-K1	NM_001001892	histocompatibility 2, K1, K region	1.5590	6.0320
H2-L	NM_001267808	histocompatibility 2, D region locus L	1.5186	5.3589
H2-Q2	NM_010392	histocompatibility 2, Q region locus 2	1.8825	5.3471
H2-Q8	NM_023124	histocompatibility 2, Q region locus 8	1.8041	5.1148
H2-T23	NM_010398	histocompatibility 2, T region locus 23	1.6700	6.5374
Hck	NM_001172117	hemopoietic cell kinase	1.6163	4.2059
Hcst	NM_011827	hematopoietic cell signal transducer	2.2545	3.7062
Hdac9	NM_024124	histone deacetylase 9	2.2139	3.1437
Ifit3	NM_010501	interferon-induced protein with tetratricopeptide repeats 3	1.8637	195.3371
Ifitm1	NM_001112715	interferon induced transmembrane protein 1	1.9323	5.5506
Ihh	ENSMUST00000189364	Indian hedgehog	3.2654	5.6386
Il12rb1	NM_008353	interleukin 12 receptor, beta 1	4.0324	10.5374
Il17ra	NM_008359	interleukin 17 receptor A	1.8363	1.7790
Il17re	NM_001034031	interleukin 17 receptor E	1.6714	1.7276
Il18r1	NM_008365	interleukin 18 receptor 1	8.7951	12.7355
Il18r1	NM_008365	interleukin 18 receptor 1	8.7951	12.7355
Iqgap1	AK163056	RIKEN full-length enriched library, clone:A430107C16, IQ motif containing GTPase activating protein 1	2.1734	2.9945
Irf1	NM_008390	interferon regulatory factor 1	1.8450	3.7933
Itga6	NM_008397	integrin alpha 6	1.8872	2.3791
Itgal	NM_008400	integrin alpha L	1.6275	2.7839
Itgb2	NM_008404	integrin beta 2	3.5450	2.4947
Itgb3	NM_016780	integrin beta 3	1.6454	5.2002
Jak3	NM_010589	Janus kinase 3	2.3181	4.6818
Jph4	NM_177049	junctophilin 4	1.8159	2.3653
Lat	NM_010689	linker for activation of T cells	1.5633	3.0780
Lcp1	NM_008879	lymphocyte cytosolic protein 1	1.5686	2.1851
Ldlr	AK161104	RIKEN full-length enriched library, clone:4732431M03, low density lipoprotein receptor	1.7895	2.3117
Lef1	NM_010703	lymphoid enhancer binding factor 1	5.3778	3.9223
Lepr	ENSMUST00000128948	leptin receptor	1.6815	1.6553
Lgals3	NM_001145953	lectin, galactose binding, soluble 3	1.8753	4.1884
Litaf	NM_019980	LPS-induced TN factor	1.5597	4.0393
Loxl3	NM_013586	lysyl oxidase-like 3	1.7393	2.3712
Lpl	NM_008509	lipoprotein lipase	1.6477	1.9784
Lrp5	AK081025	RIKEN full-length enriched library, clone:B930069L06, ow density lipoprotein receptor-related protein 5	1.5220	1.6203
Map3k5	NM_008580	mitogen-activated protein kinase kinase kinase 5	1.6519	1.6732
Mcoln2	NM_026656	mucolipin 2	3.1364	2.1144
Me2	AK080403	RIKEN full-length enriched library, clone:A630098G22, similar to NAD-DEPENDENT MALIC ENZYME, MITOCHONDRIAL PRECURSOR (EC 1.1.1.38) (NAD-ME)	1.6286	1.6249
Mef2c	ENSMUST00000196832	myocyte enhancer factor 2C	1.5611	2.0814
Milr1	NM_001271375	mast cell immunoglobulin like receptor 1	1.9466	4.8126
Mmp9	NM_013599	matrix metallopeptidase 9	2.7573	2.4887
Msn	ENSMUST00000149985	moesin	1.5099	1.5349
Muc5b	NM_028801	mucin 5, subtype B, tracheobronchial	1.5095	2.8051

Supplementary Table S3 (cont.). Microarray data of the genes included in the enriched GOs related to inflammation in Supplementary Fig. 2

Gene symbol	Accession #	Gene name	*Fold change*	
			**tg1**	**tg2**
Myd88	NM_010851	myeloid differentiation primary response gene 88	1.5404	4.4257
Myo1e	NM_181072	myosin IE	1.6896	2.5381
Nfkbiz	NM_030612	nuclear factor of kappa light polypeptide gene enhancer in B cells inhibitor, zeta	1.9690	3.3608
Ocstamp	NM_029021	osteoclast stimulatory transmembrane protein	1.5172	1.6163
Osmr	NM_011019	oncostatin M receptor	1.7709	4.0955
Panx3	NM_172454	pannexin 3	2.4537	2.8327
Pawr	NM_054056	PRKC, apoptosis, WT1, regulator	1.7485	1.7097
Pcolce2	NM_029620	procollagen C-endopeptidase enhancer 2	1.9786	2.3626
Pdgfb	AK087840	RIKEN full-length enriched library, clone:E330027M19, platelet derived growth factor, B polypeptide	1.5371	1.6858
Peli1	NM_023324	pellino 1	1.5161	1.5350
Pfkp	NM_001291071	phosphofructokinase, platelet	1.9099	2.6756
Pik3r1	NM_001077495	phosphatidylinositol 3-kinase, regulatory subunit, polypeptide 1 (p85 alpha)	1.6276	1.8801
Pla2g7	NM_013737	phospholipase A2, group VII (platelet-activating factor acetylhydrolase, plasma)	2.3110	4.6145
Plcb1	NM_019677	phospholipase C, beta 1	1.7202	1.6712
Plcg2	NM_172285	phospholipase C, gamma 2	1.5379	1.7218
Plscr1	NM_011636	phospholipid scramblase 1	1.7528	4.2975
Prkch	NM_008856	protein kinase C, eta	2.0907	1.6390
Ptger4	NM_008965	prostaglandin E receptor 4 (subtype EP4)	1.5633	2.3569
Ptgs2	NM_011198	prostaglandin-endoperoxide synthase 2	2.1229	12.4992
Ptk2b	NM_001162365	PTK2 protein tyrosine kinase 2 beta	2.1684	4.0562
Ptprc	ENSMUST00000185570	protein tyrosine phosphatase, receptor type, C	2.7270	6.4030
Ptpre	NM_011212	protein tyrosine phosphatase, receptor type, E	1.5024	1.5717
Ptprj	NM_001135657	protein tyrosine phosphatase, receptor type, J	1.7486	4.6399
Ripk2	NM_138952	receptor (TNFRSF)-interacting serine-threonine kinase 2	1.9423	3.4161
Runx1	NM_009821	runt related transcription factor 1	2.6409	4.3748
Runx2	NM_009820	runt related transcription factor 2	2.3827	3.0484
Runx3	NM_019732	runt related transcription factor 3	3.0934	2.0285
Sbno2	BC064113	strawberry notch homolog 2 (Drosophila),	1.7381	6.2630
Scin	NM_009132	scinderin	2.4570	2.6279
Sema7a	NM_011352	sema domain, immunoglobulin domain (Ig), and GPI membrane anchor, (semaphorin) 7A	2.4327	4.1282
Serinc5	AK163675	RIKEN full-length enriched library, clone:B930007O12, hypothetical TMS membrane protein/tumour differentially expressed protein/Cysteine-rich region profile/2Fe-2S ferredoxin, iron-sulfur binding site containing protein	2.1731	3.8011
Serpina3n	NM_009252	serine (or cysteine) peptidase inhibitor, clade A, member 3N	31.4082	138.7072
Slpi	NM_011414	secretory leukocyte peptidase inhibitor	5.4264	39.9103
Smpd3	NM_021491	sphingomyelin phosphodiesterase 3, neutral	1.7497	3.3005
Snx10	NM_028035	sorting nexin 10	1.9266	1.8202
Sox6	NM_011445	SRY (sex determining region Y)-box 6	2.1130	2.0565
Sox9	NM_011448	SRY (sex determining region Y)-box 9	2.8803	2.1901
Sp7	NM_130458	Sp7 transcription factor 7	2.2511	3.9924
Spp1	NM_009263	secreted phosphoprotein 1	3.3440	10.0947
Stat4	NM_011487	signal transducer and activator of transcription 4	1.8237	2.5008
Tcf7	ENSMUST00000086844	transcription factor 7, T cell specific	1.5152	2.0899
Tcirg1	NM_016921	T cell, immune regulator 1, ATPase, H+ transporting, lysosomal V0 protein A3	1.5052	1.6404
Tfeb	NM_001161722	transcription factor EB	2.3501	6.9477
Tfrc	NM_011638	transferrin receptor	2.0221	1.9583
Tgfb1	NM_011577	Mus musculus transforming growth factor, beta 1	1.9206	3.9865
Tgtp2	NM_001145164	T cell specific GTPase 2	1.7509	91.2704
Tifa	NM_145133	TRAF-interacting protein with forkhead-associated domain	1.6110	3.2450
Timp1	NM_001044384	tissue inhibitor of metalloproteinase 1	2.8625	5.4598
Timp4	NM_080639	tissue inhibitor of metalloproteinase 4	2.7520	4.6340
Tmem173	NM_028261	transmembrane protein 173	1.8083	4.6743
Tnfrsf13b	NM_021349	tumor necrosis factor receptor superfamily, member 13b	1.5137	1.6744
Tnfrsf18	NM_009400	tumor necrosis factor receptor superfamily, member 18	2.0218	2.3805
Tnfrsf1b	NM_011610	tumor necrosis factor receptor superfamily, member 1b	1.5727	5.8719
Tnfsf9	NM_009404	tumor necrosis factor (ligand) superfamily, member 9	1.7874	2.5648
Tox	ENSMUST00000150082	thymocyte selection-associated high mobility group box	2.3570	1.8988
Trim12a	NM_023835	tripartite motif-containing 12A	1.8128	1.8328
Trim34a	NM_030684	tripartite motif-containing 34A	1.6414	3.4226
Trpv4	NM_022017	transient receptor potential cation channel, subfamily V, member 4	2.3911	3.7284
Tyrobp	NM_011662	TYRO protein tyrosine kinase binding protein	1.6386	2.4356
Tyrobp	NM_011662	TYRO protein tyrosine kinase binding protein	1.6386	2.4356
Vegfa	NM_001025250	vascular endothelial growth factor A	3.0173	2.3642
Zc3hav1	NM_028864	zinc finger CCCH type, antiviral 1	1.6748	4.3624
Zfp64	NM_009564	zinc finger protein 64	1.7883	2.0163
Zmiz1	ENSMUST00000159766	zinc finger, MIZ-type containing 1	1.8225	1.5070

GENE SYMBOL	Taqman probes
Runx2	Mm00501584_m1
Hck	Mm01241463_m1
Lef1	Mm00550265_m1
Tcf7	Mm00493445_m1
Sp7	Mm04933803_m1
Dkk1	Mm00438422_m1
Col2a1	Mm01309565_m1
Gli1	Mm00494654_m1
Ihh	Mm00439613_m1
Sox9	Mm00448840_m1
Acan	Mm00545794_m1
Col10a1	Mm00487041_m1
Actb	Mm02619580_g1

SYBR Green Assays

	Fwd. Primer	Rev. Primer
Hck	5'-GCTACATCCCAAGCAACTAT-3'	5'-AAAGTCTCGAACAGACAACG-3'
Actb	5'-CCACCCGCGAGCACAGCTTC-3'	5'-TTGTCGACGACCAGCGCAGC-3'

ChIP primers

	Fwd. Primer	Rev. Primer
#1	5’-AGCCCCCTACGAGACCTTAA-3’	5’-ATGCCTACATGCACGCGTAA-3’
#2	5’-ATCCTCGCAGCTAGACCGA-3’	5’-GGTTTGCATCACCCAGGGT-3‘

Supplementary Table 4.

The primer sequences and TaqMan probes in real-time RT-PCR analyses and the primer sequences in the ChIP assay.
